# Similar minds age alike: an MRI similarity approach for predicting age-related cognitive decline

**DOI:** 10.1038/s41514-026-00345-1

**Published:** 2026-02-06

**Authors:** Blanca Zufiria-Gerbolés, Jiawei Sun, Jesús Pineda, Giovanni Volpe, Mite Mijalkov, Joana B. Pereira

**Affiliations:** 1 Department of Clinical Neuroscience, Division of Neuro, Karolinska Institutet, Stockholm, Sweden; 2 Department of Physics, Goteborg University, Goteborg, Sweden

**Keywords:** Computational biology and bioinformatics, Neuroscience

## Abstract

As individuals age, cortical alterations in brain structure contribute to cognitive decline. However, the specific patterns of age-related changes and their impact on cognition remain poorly understood. This study assessed the effects of aging on individual gray matter similarity networks and compared them to anatomical and functional connectivity networks derived from diffusion-weighted imaging and resting-state fMRI, respectively. Our results showed that gray matter similarity networks outperformed anatomical and functional connectivity in predicting age and cognition, showing the earliest age-related changes across the adult lifespan. These networks also demonstrated greater robustness to individual differences in cognition, behavior, and sex. Notably, age-related changes in gray matter similarity were associated with the brain’s underlying cytoarchitecture, being strongest in brain regions from cortical layers II and III. These findings provide a new biological insight into the neural mechanisms of cognitive aging and highlight the potential of individual morphological similarity for capturing complex brain changes across the lifespan.

## Introduction

Aging is not a uniform process, with some individuals experiencing rapid cognitive decline, whereas others remain relatively stable into late adulthood^1,2^. Understanding the factors that drive these individual differences is a central question in cognitive aging research. Among the many mechanisms explored, brain connectivity has emerged as a key candidate, given the close relationship between the organization of brain networks and cognitive functioning^3^.

Previous studies show that both functional and anatomical brain networks, measured with diffusion-weighted imaging (DWI)^4^ and functional MRI (fMRI)^5^, change with age and are associated with declines in attention, executive abilities, memory, and other cognitive abilities^6–13^. Age-related changes have been observed in major white matter tracts, such as the corpus callosum and cingulum^14–17^, as well as in functional connectivity within the default mode^9,16,18^ and frontoparietal functional networks^19^. These changes reflect reduced directionality or increased diffusion of water molecules within white matter fibers, and decreased temporal synchronization of functional signals, respectively, leading to altered communication between brain regions. Such alterations may impair efficient cognitive and motor processing^9,20,21^. and contribute to reduced information integration across the brain. However, the extent to which these changes explain variability in age-related cognitive outcomes is limited^22–24^. This has raised questions about the sensitivity of current imaging approaches and the potential confounding effects of motion, physiological noise, and other factors associated with DWI and fMRI sequences^4,25–27^. Moreover, it remains unclear which aspects of brain organization are not captured by these widely used methods.

In response to these challenges, recent work has introduced a new class of methods that estimate the similarity between brain regions using structural MRI data, a modality that, compared to DWI and fMRI, is easier to acquire, retains a better spatial resolution and is more commonly used in the clinic^28,29^. These approaches compute correlations between regions based on features such as cortical thickness (CT), mean curvature (MC), surface area (SA), sulcal depth (SD), and gray matter volume, resulting in a “morphometric similarity network”. This type of network captures the structural resemblance between regions and has shown promise in predicting age and cognitive performance^30–35^. However, because these methods rely on a small set of often highly correlated morphometric features—regional correlations are performed between four or five morphometric values (CT, MC, SA, SD, gray matter volume)—the amount of independent information available to characterize inter-regional similarity is limited, which can prevent the detection of meaningful links between brain areas^35^. This limitation can reduce the method’s capacity to uncover significant associations between brain areas. Although recent efforts have used multivariate distributions of voxel- or vertex-level data to deal with this issue^35–37^, these approaches are computationally intensive and, therefore, cannot be widely adopted. Finally, no studies have directly compared these similarity-based networks with both anatomical and functional connectivity measures in aging, which is essential for understanding the unique contributions and limitations of each approach.

To address these limitations, here we propose a new method that uses regional gray matter volume—a simple and widely available structural MRI measure—to construct individual brain similarity networks across the adult lifespan^38^. Our approach begins by creating a group-level network from young, healthy individuals using inter-regional correlations of gray matter volume. For each older participant, we then generate a perturbed version of this network by incorporating the participant’s data into the group network and calculating the difference between this new perturbed network and the original group-level one. This yields a personalized deviation map of brain similarity that reflects how much each individual diverges from the typical young adult pattern. This method overcomes the limitations of previous approaches by capturing individualized deviations from normative brain organization, similar to normative modeling^39^, while remaining computationally efficient and using only a single, easily accessible structural measure. Importantly, inter-regional similarity is estimated across a large reference sample rather than across a small set of morphometric features, resulting in substantially higher degrees of freedom and increased sensitivity to subtle age-related changes.

In the current study, we assessed brain similarity in a large sample of cognitively normal individuals aged 18 to 88 years and compared its performance with both anatomical and functional connectivity networks derived from the same participants. Additionally, we assessed how well each method captured variability in cognitive performance across multiple domains. Our results demonstrated that the gray matter similarity networks outperformed both anatomical and functional connectivity in predicting age and cognitive decline. Furthermore, they showed the earliest age-related changes across the lifespan, revealing subtle alterations already at 32 years compared to 37 years or 57 years detected by anatomical and functional networks, respectively. We found that regions with greater cytoarchitectural similarity showed stronger age-related alterations in gray matter, providing an interpretation for our findings. Finally, we introduced a deep learning framework that combines our similarity network approach with anatomical and functional connectivity networks, significantly improving prediction accuracy compared to using any network in isolation. Together, these findings suggest that gray matter similarity is a more sensitive marker of brain aging than commonly used measures of brain connectivity, with strong potential for detecting early structural changes that may underlie vulnerability to age-related and neurodegenerative conditions.

## Results

We included 632 cognitively normal individuals aged 18 to 88 years from the Cambridge Centre for Ageing and Neuroscience (Cam-CAN) (cam-can.org) cohort who underwent diffusion-weighted imaging (DWI), resting-state functional MRI (rs-fMRI) and T1-weighted (T1W) imaging to estimate anatomical, functional and brain similarity networks, respectively. All participants completed cognitive tests spanning six distinct domains, including global fluid intelligence, memory, sensorimotor, language, emotional, and executive functions (Table 1). To replicate our findings, we included a second cohort of 201 cognitively normal participants from the Leipzig Mind-Brain-Body (LEMON) dataset (http://fcon_1000.projects.nitrc.org/indi/retro/MPI_LEMON.html) that belonged to a younger (20-35 years old) or older (59-77 years old) age group. These individuals underwent a similar brain imaging and cognitive evaluation protocol (Supplementary Table 1).

**Table 1:** Summary of characteristics and cognitive domain tests for Cam-CAN cohort

Cam-CAN cohort variables	N	Median (Min-Max)
Age (years)	632	55 (18–88)
Sex (male/female)	(313/319)	-
Education (years)	625	15 (9–39)
Fluid intelligence—Cattell’s culture fair	615	32 (11–44)
Memory—Visual short-term memory (VSTM)	632	1.79 (0–5.76)
Language—Sentence comprehension	632	709.34 (−666.95–2548.6)
Emotion regulation—Negative reappraisal	267	0 (−4.1–4.2)
Motor & Action—Force matching task	297	1.14 (−0.0926–13.291)
Executive function—Hotel task	613	269.16 (20.19–960)
Anxiety (HADS)	629	4 (0–20)
Depression (HADS)	629	2 (0–17)
Sleep (PSQI)	601	4 (0–22)

The sample size (*N*) and the median (minimum and maximum) values are displayed for each variable. Each participant underwent a series of tests intended to evaluate different cognitive and behavioral domains: fluid intelligence (Cattell’s culture fair), memory (Visual short-term memory), language (Sentence comprehension), emotion regulation (Negative reappraisal), motor and action control (Force matching task), executive function (Hotel task), anxiety, depression, and sleep. Anxiety, depression, and sleep were only present in the Cam-CAN cohort and evaluated with the Hospital Anxiety and Depression Scale (HADS) and the Pittsburgh Sleep Quality Index (PSQI).

### Brain similarity is a better predictor of aging than anatomical and functional connectivity

All networks used in this study were built using the Brainnetome atlas with 246 cortical and subcortical regions. The networks derived from DWI were obtained by applying whole-brain tractography and extracting fractional anisotropy (FA) values between these brain regions, whereas those obtained from rs-fMRI were obtained from correlations between regional time series (“Methods—Individual networks construction”). Brain similarity networks were constructed in two steps: (1) building a reference network for young individuals (below 30 years old) by calculating the Pearson correlation coefficients in gray matter volumes between pairs of brain regions, while controlling for total intracranial volume, and (2) generating a perturbed network for each participant with or above 30 years old—formed by adding the participant’s data to the reference group—and then computing its difference from the reference network. For further details, please see “Methods—Individual networks construction”.

To predict the age of the individuals from the different networks, we trained three independent Graph Neural Networks (GNNs) models. GNNs are adapted to combine the structure and properties of graphs and they are becoming the state-of-the-art for several applications related to network neuroscience^40,41^ (see “Methods—Age prediction with GNNs” for more details). To assess differences between the networks, we performed 10-fold cross-validation, repeated 100 times using randomly generated training, validation, and test datasets for each iteration (“Methods—Age prediction with GNNs”). The performance of each model was evaluated by computing the coefficient of determination (*R*^2^), Pearson correlation coefficient (*R*), mean absolute error (MAE), and mean square error (MSE) between the true and predicted ages.

**Fig. 1:**
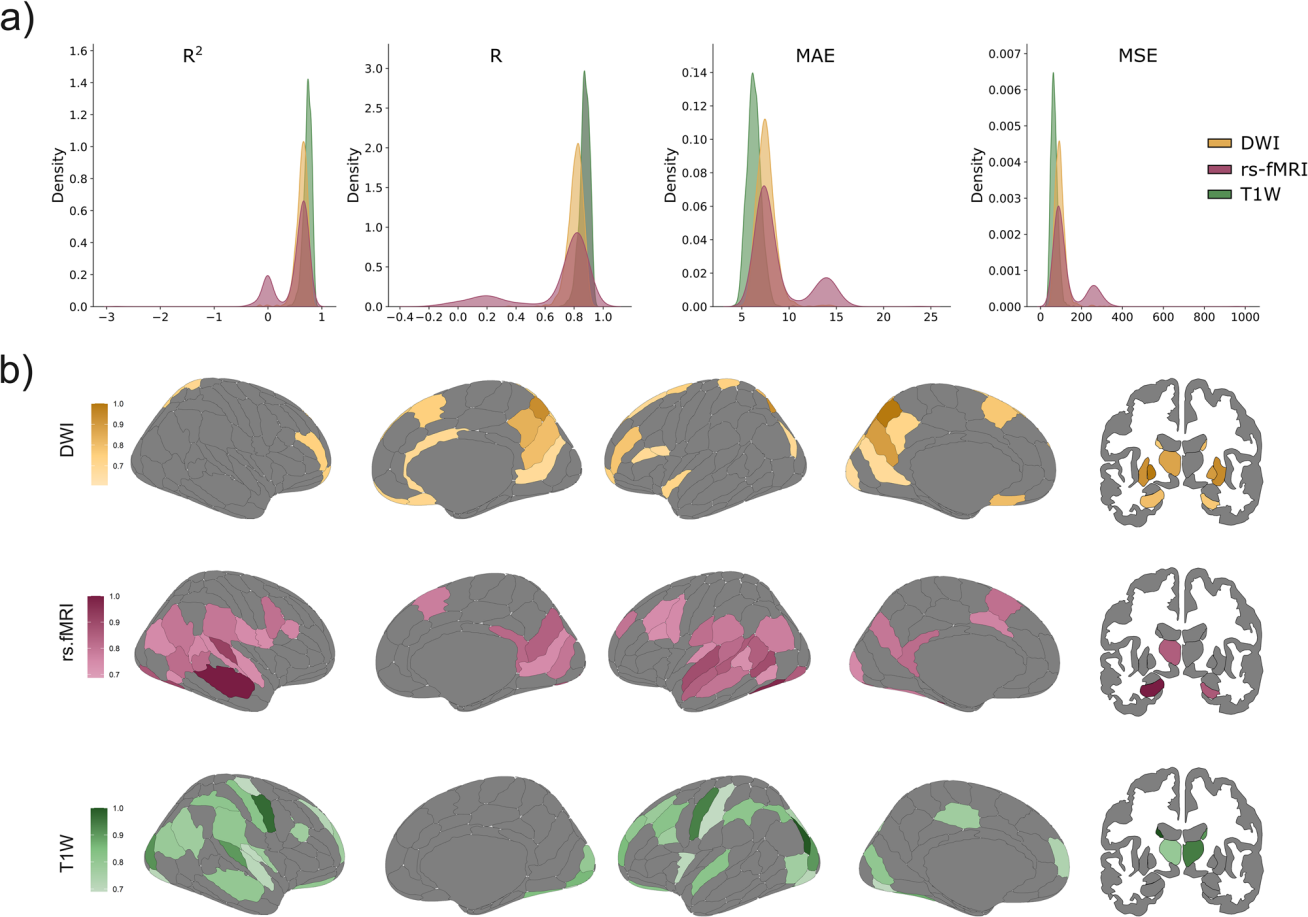
Results from the trained GNN on the different imaging modalities of the Cam-CAN cohort. **a** The performance of anatomical (DWI, yellow), functional (rs-fMRI, red) and brain similarity (T1W, green) networks is shown for four different evaluations metrics (Coefficient of determination, *R*^2^; Pearson correlation coefficient, *R*; Mean absolute error, MAE; Mean square error, MSE) between the true and predicted age with Kernel Density Estimate plots. The brain similarity model achieved the highest performance, as evidenced by highest average *R* and *R*^2^ coefficients and lowest MAE and MSE. **b** Top 20% regions that contributed the most to the prediction of age for the three different Graph Neural Network models: anatomical model from DWI, functional model from rs-fMRI and brain similarity model from T1W. DWI diffusion weighted imaging, rs-fMRI resting-state fMRI imaging, and T1W T1-weighted imaging.

Our results showed that brain similarity networks have a better age prediction compared to both anatomical (*R*: *P* = 7.70e^−20^, *R*^2^: *P* = 4.74e^−9^, MAE: *P* = 1.36e^-3^, MSE: *P* = 6.49e^−11^) and functional (*R*: *P* = 8.39e^−34^, *R*^2^: *P* = 1.36e^−35^, MAE_l_: *P* = 8.7e^−65^, MSE_l_: *P* = 8.03e^−55^) networks in the CamCAN cohort (Fig. 1a and Table 2). These results were not due to gray matter atrophy, as brain similarity also outperformed this measure in predicting age (*R*: *P* = 1.96e^−42^, *R*^2^: *P* = 2.16e^−77^, MAE: *P* = 4.91e^−99^, MSE: *P* = 8.37e^−82^) as shown in Supplementary Fig. 1. Findings were replicated in a second cohort of participants who underwent comparable brain imaging and cognitive evaluation protocols. However, participants’ ages are not continuously distributed and, grouped into a young group and an old group, require age to be modeled as a binary outcome. As a result, classification analyses were performed instead of continuous age regression. Accordingly, classification-based performance metrics (accuracy, sensitivity, specificity, and area under the curve (AUC)) were used to evaluate model performance. Similar results were found in the LEMON cohort where brain similarity networks outperformed anatomical connectivity in accuracy (*P* = 0.025) and specificity (*P* = 0.0024) and functional connectivity in accuracy (*P* = 1.09e^−90^), area under the curve (*P* = 3.5e^−86^), sensitivity (*P* = 4.53e^−35^) and specificity (*P* = 3.37e^−80^), as well as gray matter atrophy in accuracy (*P* = 4.22e^−101^), area under the curve (*P* = 1.55e^−29^), sensitivity (*P* = 4.84e^−39^) and specificity (*P* = 1.9e^−88^) when classifying young and old individuals (Supplementary Table 2 and Supplementary Fig. 1).

**Table 2:** Performance metrics across different types of models in the Cam-CAN cohort

Modality	*R*^2^	*R*	Mean absolute error (MAE)	Mean square error (MSE)
**DWI**	0.72 ± 0.083	0.80 ± 0.052	6.59 ± 0.803	70.12 ± 20.096
**rs-fMRI**	0.61 ± 0.266	0.76 ± 0.238	7.66 ± 2.669	99.71 ± 69.749
**T1W**	0.74 ± 0.079	0.87 ± 0.034	6.18 ± 0.744	64.97 ± 17.863
**DWI—rs-fMRI—T1W**	0.75 ± 0.082	0.88 ± 0.04	6.14 ± 0.865	64.01 ± 20.429

Mean and standard deviation values of the following performance metrics: Coefficient of determination (*R*^2^), Pearson correlation coefficient (*R*), Mean absolute error (MAE) and Mean square error (MSE) between the true and predicted ages. The metrics are calculated on test datasets for the 10-fold cross validation over 100 random iterations for the different types of models: diffusion-weighted imaging (DWI), resting-state functional MRI (rs-fMRI), T1W and multimodal. Results highlighted in bold show significant (*P* < 0.05) best performance.

### Brain similarity changes earlier over the course of aging than anatomical and functional connectivity

To determine which type of brain network exhibits the earliest age-related alterations, we computed four global network measures—clustering coefficient, path length, global efficiency, and modularity. These measures capture key properties of network topology, such as local segregation, global integration, and community structure, offering a more comprehensive view of network dynamics compared to simple measures such as connection strength^42^. Network measures were modeled using cubic smoothing spline models^43–45^ and the inflection age at which age-related changes start to emerge was calculated from the spline-derived first derivative. Our results revealed that brain similarity networks showed the first detectable changes for all measures: global efficiency (*P* = 32.9 years; 95% bootstrapped CI [1.0e-04,0.0065]), clustering coefficient (32.72 years; 95% bootstrapped CI [1.28e-04,0.0058]), modularity (34.12 years; 95% bootstrapped CI [−0.0507,−5.027e-04]) and path length (32.72 years; 95% bootstrapped CI [-1.46,-0.0058]). Early age-related changes in brain similarity networks are followed by anatomical connectivity networks: global efficiency (43.12 years; 95% bootstrapped CI [−0.0075,-2.14e-04]), clustering coefficient (42.88 years; 95% bootstrapped CI [−0.0066,−2.04e-04]), modularity (37.48 years; 95% bootstrapped CI [−0.0047,−1.94e-05]) and path length (43.12 years; 95% bootstrapped CI [2e-04,0.16]). Finally, functional connectivity networks are the last to show changes with aging: global efficiency (62.74 years; 95% bootstrapped CI [−0.0097,−1.9 e-04]), modularity (57.46 years; 95% bootstrapped CI [−0.0137,−1.17e-05]), modularity (71.8 years; 95% bootstrapped CI [2.85e-04,0.0154]) and path length (62.91 years; 95% bootstrapped CI [0.0047,0.1653]) (Supplementary Fig. 2). Our results align with previous aging studies, reporting white matter integrity reductions starting around 50 years old^21,46^ and functional connectivity changes from 60 years old^47^, while morphometric similarity networks were previously reported to show earlier age-related changes around 30 years old^48,49^. Finally, this pattern is consistent with lifespan developmental research, which shows that brain similarity networks are more sensitive to age-related changes than anatomical^35^ or functional^49^ connectivity networks. Together, these findings suggest that brain similarity networks detect earlier and more subtle age-related changes, both during development and aging, suggesting they are more sensitive to alterations occurring across the lifespan.

### Regional brain similarity changes show higher overlap with functional connectivity changes

To further investigate common patterns of age-related degeneration across the different network types, we analyzed the contribution of the edges (edge feature importance) to the trained GNNs. For visualization and interpretation, edge feature importance was summarized at the regional level (nodal), focusing on the top 20% most affected regions (Fig. 1b).

Brain similarity networks exhibited widespread age-related changes, affecting occipital, parietal, and temporal cortices, as well as the bilateral thalamus and caudate nuclei. Anatomical networks showed prominent changes in frontal areas, including the superior, medial, and orbital gyri, as well as the precuneus and thalamus. In contrast, functional connectivity was primarily affected in temporal regions.

Interestingly, when we computed the overlap across modalities, we observed that 26.92% of the most age-affected regions were shared between brain similarity networks and functional connectivity. Overlaps between brain similarity and anatomical networks (15.38%) and between anatomical and functional networks (7.69%) were also observed, but much smaller (Fig. 1b). Finally, the LEMON dataset showed similar patterns of overlap across networks, with brain similarity showing the highest overlap with functional connectivity networks (Supplementary Fig. 3).

### Failure to predict age is associated with different individual characteristics across distinct networks

As no model achieved 100% performance in predicting age, we investigated whether this was related to specific participant features. After correcting for age-related bias in the predicted values (for more details, see “Methods—Phenotype failure estimation”), we assessed the relationship between each model’s prediction error (computed per individual) and a range of sociodemographic (education and gender), cognitive evaluations (fluid intelligence, memory, sensorimotor abilities, language, emotion, and executive functions), psychiatric/behavioral measures (depression, anxiety and sleep quality), and white matter hyperintensities (reflecting common age-related vascular changes). The correlations between prediction errors with certain participant characteristics allows to identify possible biases in the models. When certain participant features are associated with larger prediction errors, it indicates that the model’s failure is linked to specific phenotypes. This suggests that the model’s performance in predicting age is biased towards participants with certain features, pointing to limitations in how the model captures age-related variability. Our results showed that women were estimated to be younger compared to men in the anatomical (*P* = 0.002) network model. Failure in the functional network model was associated with fluid intelligence, such that underestimated ages were predicted in those participants that best performed in this test (*R* = 0.116, *P* = 0.0073). No associations were found between the failure to predict age in the brain similarity network model with sociodemographic variables, cognitive, psychiatric, or other behavioral evaluations. However, we did find that more severe white matter hyperintensities, one of the most important changes occurring in aging^50^, affected the prediction of all the models, causing an overestimation of the predicted age that was highest in the functional networks (*R* = 0.14, *P* = 0.001), followed by anatomical (*R* = 0.13, *P* = 0.002) and finally the brain similarity (*R* = 0.12, *P* = 0.004) networks.

### Brain similarity is a key predictor of cognition and behavior

**Fig. 2:**
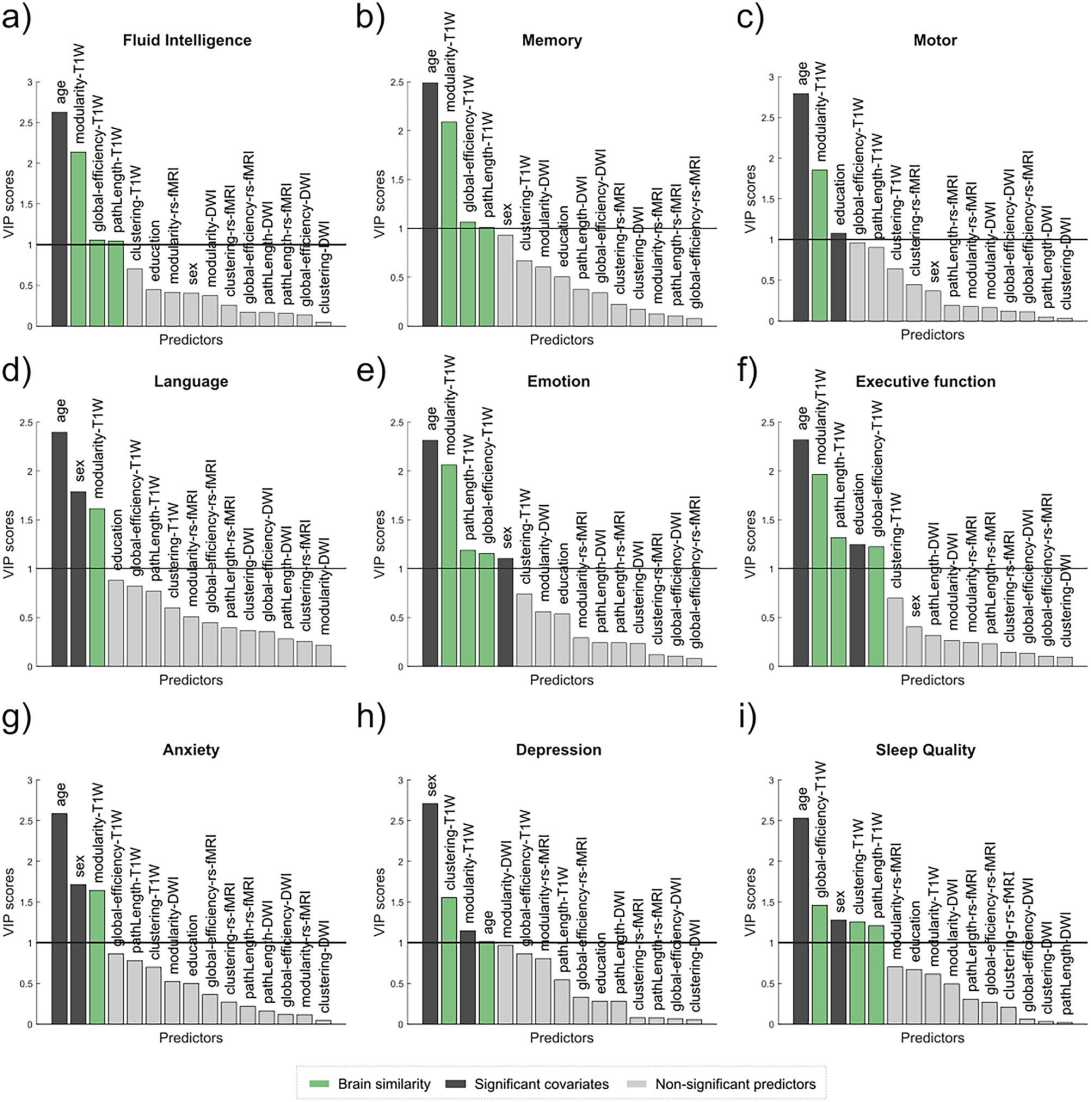
Association between the different types of networks with cognition and behavior in the Cam-CAN cohort. An independent Partial least square (PLS) model was computed for each of the following cognitive and behavioral domains: **a** Fluid intelligence, **b** Memory, **c** Motor Control, **d** Language, **e** Emotion regulation, **f** Executive function, **g** Anxiety, **h** Depression and **i** Sleep quality. Four network measures (global efficiency, path length, clustering coefficient, and modularity) derived from diffusion-weighted imaging (DWI), resting-state functional MRI (rs-fMRI), and brain similarity (T1W) networks together with age, sex and education were included as predictors in the model. Significant predictors, selected if the variable of importance (VIP) is higher than one, are colored filled, while non-significant predictors (VIP < 1) are colored filled in gray. Network measures calculated on brain similarity networks emerged as the most significant predictors (colored in green). Notably, the modularity (followed by global efficiency and path length) was a key predictor.

To compare the power of functional, anatomical and similarity-based networks to predict cognitive, motor and emotional performance, we used Partial Least Squares (PLS) models. Different tests were selected as the response of the PLS models from both Cam-CAN and LEMON cohorts covering six different cognitive domains (fluid intelligence, memory, language, emotion, motor and executive function) and three behavioral measures (anxiety, depression and sleep quality) as shown in Table 1. To avoid overfitting and improve interpretability, we extracted scalar features by computing four network measures (clustering coefficient, path length, global efficiency and modularity) on each of types of networks, and included them as predictors together with age, sex and education. Predictors were considered important if their Variable Importance in Projection (VIP) score was greater than 1^51^. The results revealed that measures calculated on brain similarity networks (especially modularity, followed by global efficiency and path length), emerged as the most significant predictors (VIP > 1) of performance in the different cognitive and behavioral tests (Fig. 2). In contrast, measures calculated on anatomical and functional networks did not emerge as significant predictors of any of the tests. These findings were replicated in the LEMON cohort, where cognition and behavior were best explained (VIP > 1) by network measures calculated on brain similarity networks, followed by functional connectivity measures (in memory and motor tests), as shown in Supplementary Fig. 4.

### Brain similarity is higher between the same cytoarchitecture cortical layers

**Fig. 3:**
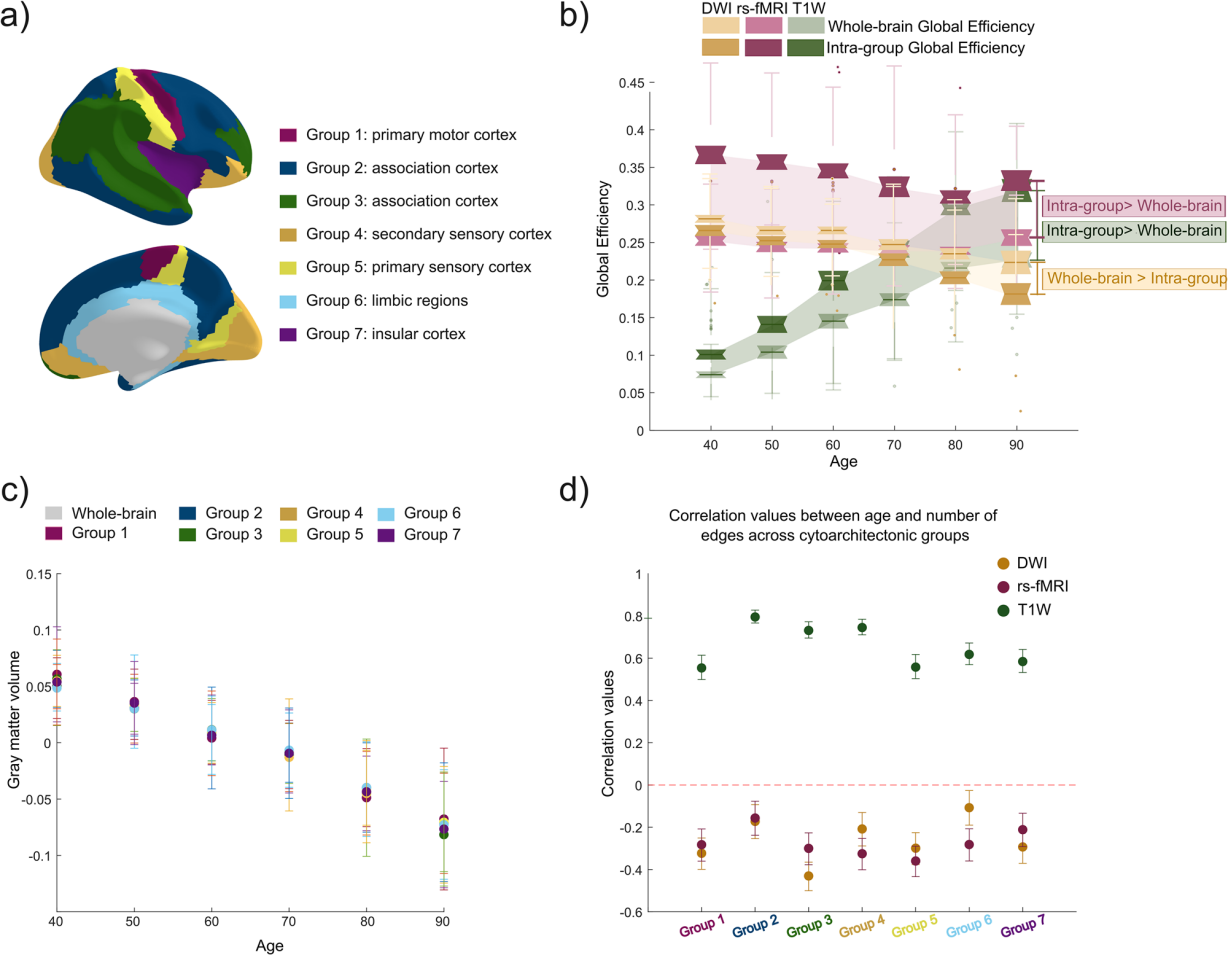
Cytoarchitecture analysis in anatomical, functional and brain similarity networks in the Cam-CAN cohort. **a** Cytoarchitecture atlas built from the mapping of Brainnetome brain regions to the five fundamental cortical types defined by von Economo and Koskinas, including the insula and cingulate cortex. **b** Global efficiency was computed across the three types of networks, both at the whole-brain level and within each cytoarchitectonic group (Intra-group). Intra-group global efficiency values were averaged across the seven cytoarchitectonic groups. **c** Changes in gray matter volumes across age for both the whole brain and each cytoarchitectonic group. No significant differences were found on gray matter atrophy between the whole brain and the different cytoarchitectonic groups. **d** Correlation values between age and number of edges in each cytoarchitectonic group. For brain similarity, the association cortex showed the strongest age-related effects, while the primary sensory cortex was the most affected by age in anatomical and functional networks.

Due to increasing evidence showing that measures of brain similarity may reflect, at least in part, the underlying cortical cytoarchitecture^52,53^, we mapped the Brainnetome atlas to the five fundamental cortical types, defined by von Economo and Koskinas^54^, including the insula and cingulate cortex^55^ (Fig. 3a). Then, we compared the global efficiency across anatomical, functional and brain similarity networks at different ages, considering both whole-brain and intra-group edges—those within the same cytoarchitectonic group. Results showed that the global efficiency calculated in brain similarity networks increased with age, whereas it decreased in both anatomical and functional networks. This pattern was observed in both whole-brain and intra-group networks (Fig. 3b). However, intra-group global efficiency was higher than whole-brain global efficiency in both gray matter similarity and functional networks across the age span. This suggests that regions within the same cytoarchitectonic group experience similar rates of gray matter similarity changes, and they are also functionally more connected. Our findings indicated that gray matter atrophy did not depend on cytoarchitectural divisions (Fig. 3c), unlike brain similarity. Finally, by exploring the correlation coefficients between age and the number of edges in each cytoarchitectonic group, we observed that while the association cortex group was the one most affected by age in brain similarity networks, the primary sensory cortex showed the strongest age-related effects in anatomical and functional connectivity (Fig. 3d).

### Integrating brain similarity, functional, and anatomical networks improves age prediction and reveals network interactions

**Fig. 4:**
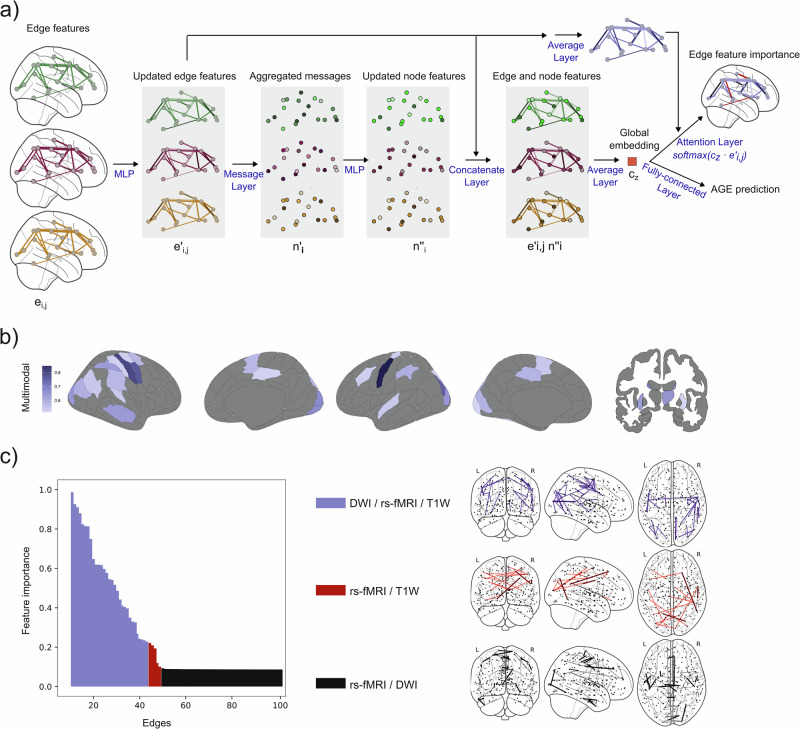
Multimodal GNN model improves the prediction of age in the Cam-CAN cohort. **a** Architecture of the multimodal model that combined information from the three types of networks derived from DWI, rs-fMRI and T1W. The model processed edge values (*e*_i,j_) form the different networks using a multilayer perceptron (MLP), then aggregated the updated edges (*e*’_i,j_) into nodes (*n*’_i_) and updated the node features with another MLP. These updated edge (*e*’_i,j_) and node features (*n*”_i_) were concatenated (*e*’_i,j_, *n*”_i_) and summarized into a global embedding (*c*_z_) for age prediction via a fully connected layer and edge-level feature importance derived using a softmax-activated attention layer. **b** Top 20% regions that contributed the most to the prediction of age for the multimodal model. **c** Edge feature importance from the multimodal model (DWI/rs-fMRI/T1W) for the 100 most contributing edges. The distinct patterns of overlapping rs-fMRI/T1W (red) and rs-fMRI/DWI (black) connections provide further insights into the brain similarity-functional and anatomical-functional relationships.

When we combined all three types of networks (similarity, anatomical, and functional) into a multimodal GNN model, we observed that a greater predictive performance was achieved, surpassing the similarity networks model alone (*R*: *P* = 5.07e^−07^, *R*^2^: *P* = 6.39e^−04^, MAE: *P* = 0.0123, MSE: *P* = 0.0037), as shown in Table 2. By exploring the edge feature importance of the multimodal model we observed that 75% of the most contributing regions were shared with brain similarity networks, suggesting that brain similarity is the most important network in the multimodal model (Fig. 4a). Further analysis of the multimodal model revealed that edges shared across the three network types were the most predictive of age, forming a bilateral pattern with clusters in the parietal and occipital lobes and an interhemispheric connection linking parietal regions. Notably, functional connections overlapping with brain similarity also exhibited strong predictive value, followed by those overlapping between functional and anatomical networks (Fig. 4b). Interestingly, the overlap between functional and brain similarity networks was primarily characterized by inter-lobe connections, whereas the overlap between functional and anatomical networks was dominated by intra-lobe connections.

Finally, in the LEMON dataset, the multimodal model did not outperform single-modality networks, likely due to their already high predictive performance. However, it showed brain patterns consistent with those observed in the CamCAN dataset. Notably, it revealed that patterns of regional overlap with brain similarity networks and edges across all three network types were the most predictive of age. Additionally, functional connections overlapping with both brain similarity and anatomical networks also showed strong predictive value in the multimodal model (Supplementary Fig. 3).

## Discussion

In recent years, increasing attention has been devoted to understanding the variability in how individuals age and why some people experience cognitive decline while others remain normal. This growing interest stems from the rising prevalence of age-related disorders and the urgent need to identify early markers that can detect deviations from normal brain aging^56^. Such markers are essential to apply timely and effective interventions before cognitive deterioration becomes irreversible. In this study, we introduced brain similarity as a novel and sensitive marker of aging and age-related cognitive decline. Compared to traditional anatomical and functional connectivity measures, brain similarity changed earlier while also providing better predictions of age, cognition, and behavior across two independent cohorts. This opens new opportunities in cognitive aging research, offering an accessible and robust tool to identify individuals at risk for cognitive decline earlier than previously possible.

Cognition is widely recognized as a distributed process that relies on interactions between brain regions. Numerous studies have demonstrated that efficient communication within networks such as the default mode network or the integrity of white matter tracts connecting Broca’s and Wernicke’s areas are essential for higher-order functions^17,57^. However, aging disrupts these interregional interactions, leading to more fragmented and less efficient communication across the brain. This fragmentation is often reflected in slower information processing and cognitive decline. Notably, most of the current understanding of age-related changes in brain connectivity and their associations with cognitive decline has been obtained from studies employing functional and diffusion MRI^9^. While functional and diffusion MRI have provided valuable insights into these changes, they are constrained by several limitations. These modalities are technically demanding, prone to noise, and more challenging to implement clinically due to their longer acquisition times, preprocessing complexity, and ongoing methodological controversies (e.g., global signal regression in rs-fMRI)^4,25–27^. In contrast, T1W MRI is routinely acquired in clinical settings and offers a structural overview of the brain that includes atrophy and structural abnormalities. Attempts to derive networks from T1W scans have often been criticized for relying on group-level analyses and for the ambiguous interpretation of correlations between morphological measures. Recent approaches that compute individual morphological networks have addressed some of these limitations by providing person-specific metrics and improving interpretability^28^. However, these methods often require computationally intensive procedures and remain statistically limited in many cases.

To overcome these barriers, in this study, we propose a simple and efficient approach to compute brain similarity from T1W images. This framework quantifies how much an individual's brain deviates from a reference cohort of young adults, making it particularly suited to aging research. Aging can be viewed as a progressive departure from youthful brain organization, and our method captures this deviation at the individual level. Across two independent datasets, our study revealed that brain similarity is a better predictor of age than anatomical and functional connectivity, only being outperformed by the multimodal model. While failure to predict age from functional and anatomical connectivity models was associated with sociodemographic variables, cognitive evaluations and white matter hyperintensities, the brain similarity model showed a more robust performance, where its failures were only associated with white matter abnormalities but still to a lower extent than anatomical or functional network models. Notably, brain similarity networks exhibited earlier changes across aging, starting at 32 years of age, compared to anatomical (37 years of age) and functional (57 years of age) connectivity. There was also an overlap of age-affected regions between brain similarity networks and functional connectivity. These findings align with previous research showing that structural covariance patterns are related to resting-state fMRI connectivity^58,59^. Furthermore, multimodal analysis revealed that edges shared across all three network types contributed the most to the prediction of age. Interestingly, functional and anatomical connectivity overlaps were characterized by intra-lobe connections, while the overlapping edges between brain similarity and functional connectivity tended to span inter-lobe connections. This phenomenon may be explained by the fact that both anatomical and functional brain connectivity decrease with the distance between regions^60,61^ and, as previous studies reported, there is a high overlap of anatomical and functional connectivity in adjacent cortical regions^61,62^. Conversely, it has been previously shown that there is a higher overlap between brain similarity and functional networks^58,59^ and that inter-lobe connections appear to be affected in age-related brain disorders^61^.

Interestingly, our analysis also identified that brain similarity networks are a better predictor of cognition, suggesting that brain similarity could detect cognitive changes earlier than anatomical and functional connectivity. Finally, although there is ongoing debate about the biological significance of brain similarity^52,63^, our results showed that cytoarchitectural characteristics are associated with brain similarity, aligning with previous literature^29^. However, these characteristics were not associated with gray matter atrophy. Thus, age-related changes in brain similarity appear to be influenced by underlying brain cytoarchitectural organization—patterns that are not fully captured by gray matter volume changes alone. Furthermore, brain similarity showed most of the age-related changes in the association cortex, while anatomical and functional connectivity are most affected by age in the primary sensory cortex. The association cortex is mainly formed by layers II and III of the cytoarchitectonic lamina of human neocortex^64^, which are evolutionarily younger, more susceptible to age-related degeneration, and among the first to deteriorate during aging^65^. These layers are also involved in cognitive processes such as memory and attention. Therefore, age-related alterations in brain similarity may reflect early cytoarchitectonic changes that directly impact cognitive function, as we have shown with our results. This finding aligns with a previous study that showed associations between differences in laminar thickness and cortical hierarchy in the human brain and interregional morphological relations, indicating that brain similarity may represent fundamental cytoarchitectural patterns in the cortex^53^. A subsequent article investigated the symmetrical and asymmetrical variations in brain similarity among brain areas, revealing that regions with comparable cytoarchitectural characteristics typically display enhanced morphological similarity associations^52^.

This study has some limitations that need to be considered. First, the analyses were performed on cross-sectional data from two independent cohorts, which does not allow capturing the longitudinal progression of age at an individual level. Future studies should aim to replicate these findings using longitudinal datasets, providing a more detailed picture of how the brain changes with age within each individual. Second, while the Cam-CAN cohort provides continuous data across the adult lifespan, the LEMON cohort provides data for non-continuous age groups. Moreover, the relatively small sample size of the LEMON cohort may have limited the statistical power of the replication results. Validation of these findings in larger, independent cohorts is needed to understand the generalizability of the results.

Despite these limitations, our findings revealed that brain similarity consistently outperformed both functional and anatomical networks in predicting chronological age and cognitive scores, establishing a new benchmark in the field. The significant overlap of key regions between brain similarity networks and functional connectivity underscores the closer relationship between these modalities. We identified that brain similarity aligns with cytoarchitectural divisions—while changes in gray matter volume occur independently of these divisions—suggesting a biological interpretation for brain similarity networks. These findings highlight the importance of brain similarity networks in understanding the neural basis of cognitive aging and its alignment with underlying biological patterns.

## Methods

### Cam-CAN cohort

A total of 632 cognitively normal individuals between 18 and 88 years old were included from the Cam-CAN dataset (cam-can.org). This study adheres to the Helsinki Declaration and has received approval from the local ethics committee, specifically the Cambridgeshire 2 Research Ethics Committee (reference^10^:/H0308/50). The exclusion criteria cover MR safety contraindications (e.g., pacemakers), mobility problems, substance abuse (past or current treatment for drug abuse or drug use), medical problems (e.g., dementia diagnosis, head injury or serious psychiatric conditions), learning disabilities (living at home), communication difficulties (e.g., hearing problems or vision difficulties), cognitive impairment (Mini-Mental State Examination (MMSE) score of 24 or lower), and reduced responses from individuals with significant longstanding illness or disability^66^. All participants underwent cognitive and behavioral assessments (Table 1).

The evaluated cognitive domains included fluid intelligence (Cattell’s Culture Fair)^67,68^, memory (Visual Short-Term Memory)^66^, language (sentence comprehension)^66^, emotional regulation (negative reappraisal)^69–71^(only obtained for a subgroup of participants^66,72^), motor skills (Force matching task)^66^, and executive functions (Hotel-task)^66,73^. To evaluate fluid intelligence, the Cattell Culture Fair test comprised four timed subtests—series completion, classification, matrices, and conditions—where participants resolve nonverbal puzzles under differing time constraints. The visual short-term memory (VSTM) task was evaluated through a continuous color report paradigm, wherein participants briefly observe colored discs, subsequently select the recalled color from a wheel after a brief interval and indicate their confidence level by the duration of their press on the chosen color. In the language test, participants heard a sentence fragment spoken by a female voice, followed by a word articulated by a male voice, and must determine if the word suitably completes the sentence. The sentence may exhibit syntactic or semantic ambiguity, and the study assesses reaction times and rejection rates according to sentence type. For the emotional task, participants watched neutral, positive, or negative film clips, rated their emotional responses, and were asked to reappraise the content of some negative clips by reinterpreting their meaning to reduce the emotional impact. Additionally, motor control was assessed by applying a target force to the participant’s left index via a force actuator and asking them to match this force either directly by pressing on the actuator with their right index finger (finger condition) or indirectly by adjusting a linear potentiometer that controls the actuator’s force level. Finally, the Hotel task required participants to act as a hotel manager and complete five fictional tasks—writing customer bills, sorting charity money, proofreading, sorting playing cards, and alphabetizing name labels—using props laid out on a table. The behavioral assessment in the cohort evaluated anxiety, depression and sleep quality. Anxiety and depression were assessed with the hospital anxiety and depression scale (HADS)^74^, while sleep was evaluated with the Pittsburgh Sleep Quality Index (PSQI)^75^.

### LEMON cohort

The LEMON (Max Planck Institut Leipzig Mind-Brain-Body) cohort (https://fcon_1000.projects.nitrc.org/indi/retro/MPI_LEMON.html) of 226 healthy participants^76,77^ was included to replicate the main findings. This cohort comprises two groups of 154 young people (20–35 years old; 109 men, 45 women) and 72 elderly people (59–77 years old; 36 men, 36 women).

The cognitive assessments covered the same cognitive domains as for the Cam-CAN dataset (Supplementary Table 1). Fluid intelligence was evaluated with the Regensburger Wortflüssigkeits-Test (RWT), which includes semantic and phonemic word fluency. The test battery for attention assessment (TAP) evaluated both working memory capacity and reaction speed. On one hand, working memory was assessed by asking participants to press a button as quickly as possible when the number displayed on screen (ranging from 1 to 9) matched the second-to-last number they saw. On the other hand, alertness was evaluated by asking participants to press a button as quickly as possible when a cross appears on the screen, with the task conducted across four rounds containing 20 stimuli each, in two conditions (with or without an audio signal preceding the visual stimulus) and preceded by a pretest. Language was evaluated with a vocabulary test where subjects had to identify one target word from a total of 42-word sets, each consisting of a real word paired with five distractor pseudo-words, with no time limit imposed. The emotion regulation questionnaire evaluated reappraisal of emotions related to affect, relationships and well-being in general. Moreover, executive function was evaluated with the Trail Making Test (TMT), where participants must quickly connect numbers in ascending order and then alternate between numbers (1–13) and letters (A–L) while connecting them as fast as possible.

### Image acquisition

For the Cam-CAN cohort, a 3T Siemens Trio scanner with a 32-channel head coil was used to obtain DWI, resting-state fMRI and T1W sequences for all participants^66^. The following criteria were used to obtain the DWI: 30 diffusion gradient directions for the two distinct values of b (1000 s/mm^2^ and 2000 s/mm^2^; repetition time (TR) = 9100 ms; echo time (TE) = 104 ms; voxel-size = 2 mm isotropic; field- of view (FOV) = 192 × 192 mm^2^; 66 axial slices; number of averages = 1); and acquisition time of 10.2 min. For the resting-state fMRI scan, participants closed their eyes and sat for 8.4 min. A Gradient-Echo Echo-Planar Imaging (GE-EPI) sequence was used with the following parameters: 32 axial slices with 3.7 mm thickness; TR = 1970 ms; TE = 30 ms; flip angle = 78°; FOV = 192 × 192 mm^2^; and voxel-size = 3 × 3 × 4.44 mm^3^. Finally, T1W structural imaging was acquired with Magnetization Prepared RApid Gradient Echo (MPRAGE) sequence with TR = 2250 ms; TE = 2.99 ms; Inversion Time (TI) = 900 ms; flip angle = 9°; FOV = 256 × 240 × 192 mm^3^; voxel-size = 1 mm isotropic; GRAPPA acceleration factor = 2; acquisition time of 4.32 min. More details regarding the image acquisition protocols in the Cam-CAN cohort can be found in ref. ^66^.

For the LEMON cohort, DWI data were acquired using a multi-band accelerated sequence combined with in-plane GRAPPA (acceleration factor = 2). The acquisition protocol included 88 axial slices with an isotropic voxel size of 1.7 mm isotropic, 60 diffusion gradient directions at a *b*-value of 1000 s/mm^2^ and 7 additional images with a *b*-value of 0 s/mm^2^. Imaging parameters were as follows: TR = 7000 ms; TE = 80 ms; flip angle (FA) = 90°; and FOV = 220 mm. For the resting state functional MRI, participants closed their eyes and sat for 15.5 min. As for Cam-CAN cohort, a GE-EPI sequence was used with the following parameters: 64 slices with 2.3 mm thickness; TR = 1400 ms; TE = 39.4 ms; flip angle = 69°; FOV = 202 × 202 mm^2^; and voxel-size = 2.3 mm isotropic. Finally, T1W structural imaging was acquired using a MPRAGE sequence with TR = 2250 ms; TE = 2.92 ms; TI1 = 700 ms; TI2 = 2500 ms; flip angle1 = 4°; flip angle 2 = 5°; and GRAPPA acceleration factor = 3. To minimize tissue signal variability caused by B1 field inhomogeneity, a grid search was conducted over flip angles. More details regarding the acquisition protocols in the LEMON cohort can be found in refs. ^66,77,78^

### Image preprocessing

Both cohorts were preprocessed with the same pipelines across the different imaging modalities, as described below.

DWI scans were preprocessed using the FSL toolbox (fsl.fmrib.ox.ac.uk/fsl/) beginning with the correction of motion and eddy current artifacts with FSL *eddy*^79^, followed by brain-extraction.

Functional scans were preprocessed through a standard pipeline implemented in fMRIPrep (v20.2.4, fmriprep.org). After removing the first two non-steady volumes, nuisance regression of motion parameters (using the 24-parameter head motion model^80^) and confounding signals from the cerebrospinal fluid and white matter was performed. High-pass filtering with a cut-off sigma of 16.67 s considering a cutoff frequency of 0.01 Hz and a TR = 3 s was then applied. Finally, brain extraction was performed.

The preprocessing of T1W images was performed using the SPM12 software (fil.ion.ucl.ac.uk/spm/software/spm12/). After reorientation, all images were segmented into gray matter, white matter and cerebrospinal fluid. The resulting gray and white matter tissues were normalized to MNI standard space using a high-dimensional warping algorithm (DARTEL). The normalised images were modulated to preserve the total amount of gray matter volume. In addition, the total intracranial volume (ICV) of each subject was calculated as the sum of gray matter, white matter and cerebrospinal fluid, to account for differences in head size in the statistical analyses.

Finally, we used the WMH-SynthSeg tool to segment the white matter of the pre-processed T1W MRI into white matter hyperintensities and hypointensities^81^.

### Individual network construction

For all individual connectivity matrices (derived from dMRI, fMRI, and T1W data), self-edges were removed by setting the diagonal to zero. For individual matrices derived from fMRI and T1W, negative weights were set to zero to ensure a non-negative weighted graph representation, which improves the stability and interpretability of graph-theoretical measures. The matrices were then sparsified at a density of 30% and rescaled so that all edge weights ranged between 0 and 1. In functional connectivity matrices, negative correlations were set to zero due to their strong dependence on preprocessing choices. In brain similarity networks, negative weights were set to zero to focus on positive weights, which were substantially more prevalent and interpretable, and to enable the computation of global graph-theoretical measures that are typically defined for non-negative weighted networks. To assess the robustness of the results across different network densities, we additionally repeated the analyses using densities of 10% and 50%. However, for clarity and to simplify the presentation of results in the manuscript, we report findings based on the 30% density threshold.

For the anatomical networks, we applied deterministic tractography to obtain the anatomical connectivity DWI matrices of each individual. Using the batch processing tool of DSI-Studio software (dsi-studio.labsolver.org), we first fitted the diffusion tensor model to estimate fractional anisotropy (FA). Then, we ran the fiber tractography using the FA values between 246 cortical and subcortical ROIs in both hemispheres defined by the Brainnetome parcellation^19^. Finally, we obtained the connectivity matrices representing the integrity values for each white matter tract connecting the different parcellated regions in the brain.

For the functional networks, we extracted the average rs-fMRI timeseries for each brain region of the Brainnetome atlas and obtained the connectivity matrices by calculating the cross-correlations between each pair of brain regions.

In the case of brain similarity networks, previous methods investigated the use of multiple morphological features that evaluate the pairwise morphological similarity of regional feature vectors constructed from several morphometric measures, which may offer a more thorough understanding of brain structure^31,35–37,82–84^. To build brain similarity networks, we applied a recently validated method shown to effectively capture subject-level variations in structural covariance^38,85^. These analyses were implemented using the open-source BRAPH 2.0 toolbox, which provides a standardized framework for constructing individual brain similarity networks^86,87^. The implementation and codes for estimating individual morphological networks in BRAPH 2.0 is currently being prepared as a separate manuscript, where, in addition to the approach used in the present study, will include additional methods for constructing individual morphometric networks such as the Kullback-Leibler (KL) divergence^32,88–92^ and the Jensen-Shannon (JS) divergence^30,34,93–96^, along with detailed documentation and a graphical user interface to facilitate straightforward implementation by other researchers. A group-level brain similarity network was first built for a young reference group by computing partial Pearson correlation coefficients (controlling for total intracranial volume) between gray matter volumes of all pairs of brain regions. This reference network was built using 70 participants (age < 30) from the Cam-CAN cohort and 72 participants (age 20–25) from the LEMON dataset, independently. Subsequently, for each remaining participant (Cam-CAN: 562 participants of age ≥ 30, LEMON: 129 participants of age ≠ 20–25), we generated a perturbed brain similarity network by incorporating the participant’s data into the reference group. The individual brain similarity network was then derived by calculating the difference between the perturbed and reference network, followed by *z*-score normalization.

### Detection of early age-related network changes

Four global network measures (clustering coefficient, path length, global efficiency, and modularity) were computed on the individual networks to capture the properties of network’s topology^86^. The clustering coefficient counts the number of triangles present around a node. The path length is the average distance from a node to all other nodes, while the global efficiency is the average of the inverse shortest path length from a node to all other nodes. Each of these three measures is calculated globally for each individual network as the average of measures of all nodes. Finally, modularity is the extent to which a network can be divided into clearly separated communities. For each of the three modalities, the four measures were calculated independently. These computations were performed using the open-source BRAPH 2.0 toolbox, which provides a standardized framework where these measures are already implemented^86,87^. Then, to obtain a robust estimate of which modality shows earlier network alterations, the global network measures were modeled using cubic smoothing spline models^43–45^. The degree of smoothing was controlled and held constant across analyses to ensure comparability. The earliest age at which significant changes emerge was identified with the spline-derived first derivative, where confidence intervals (CI) were estimated by bootstrapping 1000 times.

### Age prediction with GNNs

Graph Neural Networks (GNNs) were built in Python (v3.8.10, python.org/) with TensorFlow (v2.7.0-gpu)^97^ and Keras. GNNs are a geometric deep learning approach that exploits the information contained in non-Euclidean structures such as graphs. These GNN models are publicly available in a GitHub repository (GNN_Age_Prediction). The different types of networks in this project (anatomical, functional, brain similarity) were represented as graphs, where brain regions serve as nodes and the links between them as edges^98^. In this project, we employed a message-passing variant in which information is propagated along the edges of a graph, allowing nodes to exchange “messages”—comprising both node and edge information—with their neighboring nodes. These messages are subsequently aggregated and updated, enabling the model to capture complex relationships between nodes^41,99,100^. The updated information is then used to generate rich representations at both the node and graph levels, facilitating downstream tasks such as classification or prediction.

The GNN architecture used in our analysis is composed of edge encoding and decoding neural networks. Both the encoder and decoder architectures are formed by multi-layer perceptron (MLP) layers of dimensions 16 and 8, with a maintained latent dimension of 16. Furthermore, to enhance the interpretability of the graph, we incorporated attention layers that updated the global embedding by leveraging the hidden states of the edge features (edge-attention), based on previous work on self-attention layers in GNN^99,101^. Attention layers improve the performance of the networks by focusing on the most relevant parts of the inputs of variable sizes to make the prediction^101^. The updated global embedding is subsequently decoded to generate age predictions. These edge-attention layers capture the associations between edge features and the final prediction, thereby providing insights into edge feature importance. Thus, our edge-attention GNN facilitates the exploration of edges based on their contributions to age prediction. Furthermore, a multimodal model was built based on the edge-attention GNN to combine three different types of networks (brain similarity, anatomical connectivity and functional connectivity). The architecture of the multimodal GNN is depicted in Fig. 4a. Finally, a multi-layer perceptron (MLP) model was independently used to predict the age of the individuals from gray matter volumes to compare the predictive power of the brain similarity networks with gray matter atrophy. The architecture of this deep-learning method consisted of four hidden layers with 8, 16, 16, and 8 nodes. The output layer in both the GNN and the MLP models used a linear activation function for regression tasks and a sigmoid activation function for classification tasks.

A total of 562 individual networks for each modality and gray matter volumes (age ≥ 30) were used to train the GNNs and the MLP, respectively, where 80% and 10% of the networks were used for training and validation, and 10% for testing. The deep learning models were trained for 200 epochs with a batch size = 64, learning rate = 0.005, Adam optimizer to minimize a mean absolute error loss or the binary cross entropy for regression or classification models, respectively. For the single-modality models, the input networks were thresholded at a density of 30% (as described in the section on Individual networks construction). The performance of the GNN models was also evaluated at densities of 10% and 50%, as shown in Supplementary Fig. 5. However, because the same conclusions were obtained across densities, and for clarity and simplicity, we focus on and report the results using the 30% density in the manuscript. In contrast, the multimodal model used a three-layer input composed of networks from each modality, thresholded at a density of 10%. Consequently, all four independent GNNs were trained with networks at the specified density level.

Significant performance differences between the single modality models and multimodal models were evaluated by performing 10-fold cross-validation, repeated 100 times with randomly generated training, validation, and test datasets for each iteration. Performances between the different types of models were then compared using two-sample t-tests.

### Cognitive associations

The association between the different individual networks on cognition was evaluated using Partial Least Squares (PLS) analysis^102^. In this method, the predictor and predicted variable matrix are linearly decomposed into latent variables (LVs) using this statistical method. The LVs are then adjusted to maximize the covariance between the resulting predictor and predicted matrix elements (factors and loadings). For every cognitive test, a different PLS model was fitted with variables such as age, sex, education, and network measures as predictors. The network measures used in the present analysis were the following: clustering coefficient, path length, global efficiency and modularity^86^. Measures were calculated for the detection of early changes with ageing as explained in the “Methods—Detection of early age-related network changes” section. Cross-validation was employed to determine the optimal number of latent variables in each PLS model. To identify significant predictors, we applied the Variable Importance in Projection (VIP) statistic, which is widely used in standard PLS analyses^51^.

### Phenotype failure estimation

To explore how the separate networks fail based on different phenotypes or patient characteristics, we used the predicted values for each test dataset in the 10-fold cross-validation and for each GNNs-trained model. Thus, we analyzed each participant’s prediction in the whole dataset, where the prediction errors from each participant’s prediction were correlated with the different phenotype measures. These correlations allow us to identify possible biases in the model’s performance towards individuals with specific phenotypes. To address the bias effect of the prediction on the response (age in this case), given their strong correlation, we implemented a correction utilized in other studies^103–107^. We first modeled the relationship between the predicted and ground truth ages using the linear equation $Y=\,\alpha \varOmega +\beta$, where *α* and *β* represent the slope and intercept. Then, the estimated parameters α and β were used to adjust the predicted age according to the formula Corrected Predicted Age = Predicted Age + ½Ω (αΩ + β). After applying this correction, we examined the correlation between the corrected prediction error and various covariates (education and sex), cognitive evaluations (fluid intelligence, memory, sensorimotor abilities, language, emotion, and executive functions) and white matter hyperintensities (a measure of vascular status).

### Cytoarchitecture correspondence analysis

Previous studies have shown that differences in laminar thickness and cortical hierarchy in the human brain are significantly associated with interregional connectivity, indicating that brain similarity may represent fundamental cytoarchitectural patterns in the cortex^52,53^. To test this hypothesis, we analyzed the cytoarchitecture of our dataset by mapping the Brainnetome atlas into the five fundamental cortical types defined by von Economo and Koskinas^64^. Furthermore, the insula and cingulate cortex were also included as two extra cytoarchitecture groups^29^. Thus, all Brainnetome brain regions were assigned to one of the seven different cortical types (Fig. 3a). To evaluate the correspondence between the networks derived from the three different modalities (DWI, rs-fMRI and T1W) and the cytoarchitecture organization of the brain, we computed the global efficiency, assessing the performance in communication between different brain regions. We calculated the global efficiency across ages using the edges in the whole brain and using the edges only between the same cytoarchitectonic groups (intra-group).

To investigate whether brain similarity reflects cytoarchitectural effects independent of gray matter volume, we compared the average gray matter volume for the whole brain with the average gray matter volume of each cytoarchitectonic group region independently. Additionally, we examined the age-related decrease in gray matter volume for the whole brain and for each cytoarchitectonic group.

## Supplementary information

Supplementary information

## Data Availability

This study utilized data from two publicly available sources: the Cambridge Centre for Ageing and Neuroscience (Cam-CAN) cohort^66^ and the LEMON (Leipzig Mind-Brain-Body) cohort^76,77^ from the Max Planck Institute (as replication cohort). The Cam-CAN dataset, which includes both imaging and behavioral data, can be accessed at camcan-archive.mrccbu.cam.ac.uk/dataaccess/. Similarly, the LEMON dataset is available for public download via an Amazon Web Services S3 bucket at: fcp-indi/data/Projects/INDI/MPI-LEMON. Additional information about the cohorts can be found at and https://fcon_1000.projects.nitrc.org/indi/retro/MPI_LEMON.html.

## References

[1] Montine, T. J. et al. Concepts for brain aging: resistance, resilience, reserve, and compensation. *Alzheimer’s. Res. Ther.* **11**, 1–3 (2019). 30857563 10.1186/s13195-019-0479-y PMC6410486

[2] Stern, Y. et al. Whitepaper: defining and investigating cognitive reserve, brain reserve, and brain maintenance. *Alzheimer’s. Dement.* **16**, 1305–1311 (2020). 30222945 10.1016/j.jalz.2018.07.219 PMC6417987

[3] Fornito, A., Zalesky, A. & Breakspear, M. The connectomics of brain disorders. *Nat. Rev. Neurosci.* **16**, 159–172 (2015). 25697159 10.1038/nrn3901

[4] Zhang, F. et al. Quantitative mapping of the brain’s structural connectivity using diffusion MRI tractography: a review. *Neuroimage* **249**, 118870 (2022). 34979249 10.1016/j.neuroimage.2021.118870 PMC9257891

[5] Khanna, N. et al. Functional neuroimaging: fundamental principles and clinical applications. *Neuroradiol. J.* **28**, 87–96 (2015). 25963153 10.1177/1971400915576311 PMC4757157

[6] Douaud, G. et al. A common brain network links development, aging, and vulnerability to disease. *Proc. Natl. Acad. Sci. USA* **111**, 17648–17653 (2014). 25422429 10.1073/pnas.1410378111 PMC4267352

[7] Mijalkov, M. et al. Sex differences in multilayer functional network topology over the course of aging in 37543 UK Biobank participants. *Netw. Neurosci.* **7**, 351–376 (2023). 37334001 10.1162/netn_a_00286 PMC10275214

[8] Lin, L. et al. Predicting healthy older adult’s brain age based on structural connectivity networks using artificial neural networks. *Comput. Methods Prog. Biomed.* **125**, 8–17 (2016). 10.1016/j.cmpb.2015.11.012 26718834

[9] Damoiseaux, J. S. Effects of aging on functional and structural brain connectivity. *Neuroimage* **160**, 32–40 (2017). 28159687 10.1016/j.neuroimage.2017.01.077

[10] Puxeddu, M. G. et al. The modular organization of brain cortical connectivity across the human lifespan. *NeuroImage* **218**, 116974 (2020). 32450249 10.1016/j.neuroimage.2020.116974

[11] Ooi, L. Q. R. et al. Comparison of individualized behavioral predictions across anatomical, diffusion and functional connectivity MRI. *NeuroImage* **263**, 119636 (2022). 36116616 10.1016/j.neuroimage.2022.119636

[12] Cabeza, R. et al. Maintenance, reserve and compensation: the cognitive neuroscience of healthy ageing. *Nat. Rev. Neurosci.* **19**, 701–710 (2018). 30305711 10.1038/s41583-018-0068-2 PMC6472256

[13] Griffa, A. et al. Structural connectomics in brain diseases. *Neuroimage* **80**, 515–526 (2013). 23623973 10.1016/j.neuroimage.2013.04.056

[14] Sullivan, E. V., Adalsteinsson, E. & Pfefferbaum, A. Selective age-related degradation of anterior callosal fiber bundles quantified in vivo with fiber tracking. *Cereb. Cortex* **16**, 1030–1039 (2006). 16207932 10.1093/cercor/bhj045

[15] Salat, D. et al. Age-related alterations in white matter microstructure measured by diffusion tensor imaging. *Neurobiol. aging* **26**, 1215–1227 (2005). 15917106 10.1016/j.neurobiolaging.2004.09.017

[16] Fjell, A. M. et al. What is normal in normal aging? Effects of aging, amyloid and Alzheimer’s disease on the cerebral cortex and the hippocampus. *Prog. Neurobiol.* **117**, 20–40 (2014). 24548606 10.1016/j.pneurobio.2014.02.004 PMC4343307

[17] Coelho, A. et al. Signatures of white-matter microstructure degradation during aging and its association with cognitive status. *Sci. Rep.* **11**, 4517 (2021). 33633204 10.1038/s41598-021-83983-7 PMC7907273

[18] Brown, C. A. et al. Distinct patterns of default mode and executive control network circuitry contribute to present and future executive function in older adults. *NeuroImage* **195**, 320–332 (2019). 30953834 10.1016/j.neuroimage.2019.03.073 PMC6536351

[19] Fjell, A. M. et al. The disconnected brain and executive function decline in aging. *Cereb. Cortex* **27**, 2303–2317 (2017). 27073220 10.1093/cercor/bhw082

[20] Gong, G. et al. Age-and gender-related differences in the cortical anatomical network. *J. Neurosci.* **29**, 15684–15693 (2009). 20016083 10.1523/JNEUROSCI.2308-09.2009 PMC2831804

[21] Zhao, T. et al. Age-related changes in the topological organization of the white matter structural connectome across the human lifespan. *Hum. Brain Mapp.* **36**, 3777–3792 (2015). 26173024 10.1002/hbm.22877 PMC6869038

[22] Sebenius, I. et al. Structural MRI of brain similarity networks. *Nat. Rev. Neurosci.* **26**, 42–59 (2025). 39609622 10.1038/s41583-024-00882-2 PMC12936990

[23] Forkel, S. J. et al. White matter variability, cognition, and disorders: a systematic review. *Brain Struct. Funct*. **227**, 529–544 (2022). 10.1007/s00429-021-02382-w PMC8844174 34731328

[24] Salama, G. R. et al. Diffusion weighted/tensor imaging, functional MRI and perfusion weighted imaging in glioblastoma—foundations and future. *Front. Neurol.* **8**, 660 (2018). 29403420 10.3389/fneur.2017.00660 PMC5786563

[25] Molloy, E. K., Meyerand, M. E. & Birn, R. M. The influence of spatial resolution and smoothing on the detectability of resting-state and task fMRI. *Neuroimage* **86**, 221–230 (2014). 24021836 10.1016/j.neuroimage.2013.09.001 PMC5736131

[26] Zaitsev, M., Maclaren, J. & Herbst, M. Motion artifacts in MRI: a complex problem with many partial solutions. *J. Magn. Reson. Imaging* **42**, 887–901 (2015). 25630632 10.1002/jmri.24850 PMC4517972

[27] Le Bihan, D. et al. Artifacts and pitfalls in diffusion MRI. *J. Magn. Reson. Imaging* **24**, 478–488 (2006). 16897692 10.1002/jmri.20683

[28] Cai, M. et al. Individual-level brain morphological similarity networks: Current methodologies and applications. *CNS Neurosci. Ther.* **29**, 3713–3724 (2023). 37519018 10.1111/cns.14384 PMC10651978

[29] Seidlitz, J. et al. Morphometric similarity networks detect microscale cortical organization and predict inter-individual cognitive variation. *Neuron* **97**, 231–247.e7 (2018). 29276055 10.1016/j.neuron.2017.11.039 PMC5763517

[30] Wang, Y. et al. Age-related differences of cortical topology across the adult lifespan: Evidence from a multisite MRI study with 1427 individuals. *J. Magn. Reson. Imaging* **57**, 434–443 (2023). 35924281 10.1002/jmri.28318

[31] Li, J. et al. Tracking age-related topological changes in individual brain morphological networks across the human lifespan. *J. Magn. Reson. Imaging* **59**, 1841–1851 (2024). 37702277 10.1002/jmri.28984

[32] Kong, X. -z. et al. Mapping individual brain networks using statistical similarity in regional morphology from MRI. *PloS ONE* **10**, e0141840 (2015). 26536598 10.1371/journal.pone.0141840 PMC4633111

[33] Mertens, N. et al. The effect of aging on brain glucose metabolic connectivity revealed by [18F] FDG PET-MR and individual brain networks. *Front. Aging Neurosci.* **13**, 798410 (2022). 35221983 10.3389/fnagi.2021.798410 PMC8865456

[34] Ruan, J. et al. Single-subject cortical morphological brain networks across the adult lifespan. *Hum. Brain Mapp.* **44**, 5429–5449 (2023). 37578334 10.1002/hbm.26450 PMC10543107

[35] Sebenius, I. et al. Robust estimation of cortical similarity networks from brain MRI. *Nat. Neurosci.* **26**, 1461–1471 (2023). 37460809 10.1038/s41593-023-01376-7 PMC10400419

[36] Li, W. et al. Construction of individual morphological brain networks with multiple morphometric features. *Front. Neuroanat.* **11**, 34 (2017). 28487638 10.3389/fnana.2017.00034 PMC5403938

[37] Yao, G. et al. Transcriptional patterns of the cortical Morphometric Inverse Divergence in first-episode, treatment-naïve early-onset schizophrenia. *NeuroImage* **285**, 120493 (2024). 38086496 10.1016/j.neuroimage.2023.120493

[38] Liu, Z. et al. Resolving heterogeneity in schizophrenia through a novel systems approach to brain structure: individualized structural covariance network analysis. *Mol. Psychiatry* **26**, 7719–7731 (2021). 34316005 10.1038/s41380-021-01229-4

[39] Ge, R. et al. Normative modelling of brain morphometry across the lifespan with CentileBrain: algorithm benchmarking and model optimisation. *Lancet Digit. Health* **6**, e211–e221 (2024). 38395541 10.1016/S2589-7500(23)00250-9 PMC10929064

[40] Wang, P. Y. et al. Generalizable machine learning in neuroscience using graph neural networks. *Front. Artif. Intell.* **4**, 618372 (2021). 33748747 10.3389/frai.2021.618372 PMC7971515

[41] Bessadok, A., Mahjoub, M. A. & Rekik, I. Graph neural networks in network neuroscience. *IEEE Trans. Pattern Anal. Mach. Intell.* **45**, 5833–5848 (2022). 10.1109/TPAMI.2022.3209686 36155474

[42] Farahani, F. V., Karwowski, W. & Lighthall, N. R. Application of graph theory for identifying connectivity patterns in human brain networks: a systematic review. *Front. Neurosci.* **13**, 585 (2019). 31249501 10.3389/fnins.2019.00585 PMC6582769

[43] Fjell, A. M. et al. Critical ages in the life course of the adult brain: nonlinear subcortical aging. *Neurobiol. Aging* **34**, 2239–2247 (2013). 23643484 10.1016/j.neurobiolaging.2013.04.006 PMC3706494

[44] Ziegler, G. et al. Models of the aging brain structure and individual decline. *Front. Neuroinformatics* **6**, 3 (2012). 10.3389/fninf.2012.00003 PMC3303090 22435060

[45] Aboud, K. S. et al. Structural covariance across the lifespan: brain development and aging through the lens of inter-network relationships. *Hum. Brain Mapp.* **40**, 125–136 (2019). 30368995 10.1002/hbm.24359 PMC6478172

[46] Bethlehem, R. A. et al. Brain charts for the human lifespan. *Nature* **604**, 525–533 (2022). 35388223 10.1038/s41586-022-04554-y PMC9021021

[47] Sun, L. et al. Human lifespan changes in the brain’s functional connectome. Nat. Neurosci. **28**, 891–901 (2025). 10.1038/s41593-025-01907-4 40181189

[48] Niu, J. et al. Age-associated cortical similarity networks correlate with cell type-specific transcriptional signatures. *Cereb. Cortex* **34**, bhad454 (2024). 38037843 10.1093/cercor/bhad454

[49] Liang, X. et al. Dissecting human cortical similarity networks across the lifespan. *Neuron* **113**, 3275–3295.e11 (2025). 40706587 10.1016/j.neuron.2025.06.018

[50] Habes, M. et al. White matter hyperintensities and imaging patterns of brain ageing in the general population. *Brain* **139**, 1164–1179 (2016). 26912649 10.1093/brain/aww008 PMC5006227

[51] Chong, I.-G. & Jun, C.-H. Performance of some variable selection methods when multicollinearity is present. *Chemom. Intell. Lab. Syst.* **78**, 103–112 (2005).

[52] Mechelli, A. et al. Structural covariance in the human cortex. *J. Neurosci.* **25**, 8303–8310 (2005). 16148238 10.1523/JNEUROSCI.0357-05.2005 PMC6725541

[53] Saberi, A. et al. The regional variation of laminar thickness in the human isocortex is related to cortical hierarchy and interregional connectivity. *PLoS Biol.* **21**, e3002365 (2023). 37943873 10.1371/journal.pbio.3002365 PMC10684102

[54] von Economo, C. F. & Koskinas, G. N. Die Cytoarchitektonik der Hirnrinde des erwachsenen Menschen. *Arch. NeurPsych.* **16**, 816 (1926).

[55] Vértes, P. E. et al. Gene transcription profiles associated with inter-modular hubs and connection distance in human functional magnetic resonance imaging networks. *Philos. Trans. R. Soc. B Biol. Sci.* **371**, 20150362 (2016). 10.1098/rstb.2015.0362 PMC5003862 27574314

[56] Jiang, Q. et al. Antiageing strategy for neurodegenerative diseases: from mechanisms to clinical advances. *Signal Transduct. Target. Ther.* **10**, 76 (2025). 40059211 10.1038/s41392-025-02145-7 PMC11891338

[57] Perovnik, M. et al. Functional brain networks in the evaluation of patients with neurodegenerative disorders. *Nat. Rev. Neurol.* **19**, 73–90 (2023). 36539533 10.1038/s41582-022-00753-3

[58] Kelly, C. et al. A convergent functional architecture of the insula emerges across imaging modalities. *Neuroimage* **61**, 1129–1142 (2012). 22440648 10.1016/j.neuroimage.2012.03.021 PMC3376229

[59] Seeley, W. W. et al. Neurodegenerative diseases target large-scale human brain networks. *Neuron* **62**, 42–52 (2009). 19376066 10.1016/j.neuron.2009.03.024 PMC2691647

[60] Ouyang, M. et al. Short-range connections in the developmental connectome during typical and atypical brain maturation. *Neurosci. Biobehav. Rev.* **83**, 109–122 (2017). 29024679 10.1016/j.neubiorev.2017.10.007 PMC5730465

[61] Honey, C. J. et al. Predicting human resting-state functional connectivity from structural connectivity. *Proc. Natl. Acad. Sci. USA* **106**, 2035–2040 (2009). 19188601 10.1073/pnas.0811168106 PMC2634800

[62] Damoiseaux, J. S. & Greicius, M. D. Greater than the sum of its parts: a review of studies combining structural connectivity and resting-state functional connectivity. *Brain Struct. Funct.* **213**, 525–533 (2009). 19565262 10.1007/s00429-009-0208-6

[63] Alexander-Bloch, A., Giedd, J. N. & Bullmore, E. Imaging structural co-variance between human brain regions. *Nat. Rev. Neurosci.* **14**, 322–336 (2013). 23531697 10.1038/nrn3465 PMC4043276

[64] Solari, S. V. H. & Stoner, R. M. Cognitive consilience: primate non-primary neuroanatomical circuits underlying cognition. *Front. Neuroanat.* **5**, 65 (2011). 10.3389/fnana.2011.00065 PMC3243081 22194717

[65] Morrison, J. H. & Hof, P. R. Life and death of neurons in the aging cerebral cortex. *Int. Rev. Neurobiol.* **81**, 41–57 (2007). 17433917 10.1016/S0074-7742(06)81004-4

[66] Shafto, M. A. et al. The Cambridge Centre for Ageing and Neuroscience (Cam-CAN) study protocol: a cross-sectional, lifespan, multidisciplinary examination of healthy cognitive ageing. *BMC Neurol.* **14**, 204 (2014). 25412575 10.1186/s12883-014-0204-1 PMC4219118

[67] Cattell, R. B. & Cattell, A. K. *Measuring Intelligence With the Culture Fair Tests*. (Institute for Personality and Ability Testing, 1960).

[68] Kievit, R. A. et al. Distinct aspects of frontal lobe structure mediate age-related differences in fluid intelligence and multitasking. *Nat. Commun*. **5**, 5658 (2014). 10.1038/ncomms6658 PMC4284640 25519467

[69] Mather, M. & Carstensen, L. L. Aging and motivated cognition: the positivity effect in attention and memory. *Trends Cogn. Sci.* **9**, 496–502 (2005). 16154382 10.1016/j.tics.2005.08.005

[70] Dalgleish, T. The emotional brain. *Nat. Rev. Neurosci.* **5**, 583–589 (2004). 15208700 10.1038/nrn1432

[71] Mather, M. The emotion paradox in the aging brain. *Ann. N. Y. Acad. Sci.* **1251**, 33–49 (2012). 22409159 10.1111/j.1749-6632.2012.06471.x PMC3395773

[72] Taylor, J. R. et al. The Cambridge Centre for Ageing and Neuroscience (Cam-CAN) data repository: Structural and functional MRI, MEG, and cognitive data from a cross-sectional adult lifespan sample. *Neuroimage* **144**, 262–269 (2017). 26375206 10.1016/j.neuroimage.2015.09.018 PMC5182075

[73] Shallice, T. & Burgess, P. W. Deficits in strategy application following frontal lobe damage in man. *Brain* **114**, 727–741 (1991). 2043945 10.1093/brain/114.2.727

[74] Zigmond, A. S. & Snaith, R. P. The hospital anxiety and depression scale. *Acta Psychiatr. Scand.* **67**, 361–370 (1983). 6880820 10.1111/j.1600-0447.1983.tb09716.x

[75] Buysse, D. J. et al. The Pittsburgh Sleep Quality Index: a new instrument for psychiatric practice and research. *Psychiatry Res.* **28**, 193–213 (1989). 2748771 10.1016/0165-1781(89)90047-4

[76] Babayan, A. et al. A mind-brain-body dataset of MRI, EEG, cognition, emotion, and peripheral physiology in young and old adults. *Sci. Data* **6**, 1–21 (2019). 30747911 10.1038/sdata.2018.308 PMC6371893

[77] Mendes, N. et al. A functional connectome phenotyping dataset including cognitive state and personality measures. *Sci. Data* **6**, 1–19 (2019). 30747913 10.1038/sdata.2018.307 PMC6371896

[78] Marques, J. P. et al. MP2RAGE, a self bias-field corrected sequence for improved segmentation and T1-mapping at high field. *Neuroimage* **49**, 1271–1281 (2010). 19819338 10.1016/j.neuroimage.2009.10.002

[79] Andersson, J. L. R. & Sotiropoulos, S. N. An integrated approach to correction for off-resonance effects and subject movement in diffusion MR imaging. *Neuroimage* **125**, 1063–1078 (2016). 26481672 10.1016/j.neuroimage.2015.10.019 PMC4692656

[80] Friston, K. J. et al. Movement-related effects in fMRI time-series. *Magn. Reson. Med.* **35**, 346–355 (1996). 8699946 10.1002/mrm.1910350312

[81] Laso, P., et al. Quantifying white matter hyperintensity and brain volumes in heterogeneous clinical and low-field portable MRI. in 2024 IEEE International Symposium on Biomedical Imaging (ISBI). 2024. IEEE. 10.1109/isbi56570.2024.10635502 PMC12369672 40851670

[82] Ding, H. et al. Topological properties of individual gray matter morphological networks in identifying the preclinical stages of Alzheimer’s disease: a preliminary study. *Quant. Imaging Med. Surg.* **13**, 5258 (2023). 37581056 10.21037/qims-22-1373 PMC10423385

[83] Yu, K. et al. Individual morphological brain network construction based on multivariate euclidean distances between brain regions. *Front. Hum. Neurosci.* **12**, 204 (2018). 29887798 10.3389/fnhum.2018.00204 PMC5981802

[84] Li, W. et al. Alterations of graphic properties and related cognitive functioning changes in mild Alzheimer’s disease revealed by individual morphological brain network. *Front. Neurosci.* **12**, 927 (2018). 30618556 10.3389/fnins.2018.00927 PMC6295573

[85] Liu, X. et al. Personalized characterization of diseases using sample-specific networks. *Nucleic Acids Res.* **44**, e164 (2016). 27596597 10.1093/nar/gkw772 PMC5159538

[86] Chang, Y.-W. et al. BRAPH 2: a flexible, open-source, reproducible, community-oriented, easy-to-use framework for network analyses in neurosciences. Preprint at bioRxiv, 10.1101/2025.04.11.648455 (2025).

[87] Mijalkov, M. et al. BRAPH: a graph theory software for the analysis of brain connectivity. *PloS ONE* **12**, e0178798 (2017). 28763447 10.1371/journal.pone.0178798 PMC5538719

[88] Wang, M. et al. Individual brain metabolic connectome indicator based on Kullback-Leibler Divergence Similarity Estimation predicts progression from mild cognitive impairment to Alzheimer’s dementia. *Eur. J. Nucl. Med. Mol. Imaging* **47**, 2753–2764 (2020). 32318784 10.1007/s00259-020-04814-x PMC7567735

[89] Wang, L. et al. A metabolism-functional connectome sparse coupling method to reveal imaging markers for Alzheimer’s disease based on simultaneous PET/MRI scans. *Hum. Brain Mapp.* **44**, 6020–6030 (2023). 37740923 10.1002/hbm.26493 PMC10619407

[90] Xu, X. et al. Morphological, structural, and functional networks highlight the role of the cortical-subcortical circuit in individuals with subjective cognitive decline. *Front. Aging Neurosci.* **13**, 688113 (2021). 34305568 10.3389/fnagi.2021.688113 PMC8299728

[91] Chen, H. et al. Alzheimer’s disease clinical scores prediction based on the label distribution learning using brain structural MRI. In *Proc*. *2022 International Joint Conference on Neural Networks (IJCNN)* (IEEE, 2022).

[92] Wang, H. et al. Single-subject morphological brain networks: connectivity mapping, topological characterization and test–retest reliability. *Brain Behav.* **6**, e00448 (2016). 27088054 10.1002/brb3.448 PMC4782249

[93] Xu, X. et al. Altered pattern analysis and identification of subjective cognitive decline based on morphological brain network. *Front. Aging Neurosci.* **14**, 965923 (2022). 36034138 10.3389/fnagi.2022.965923 PMC9404502

[94] Peng, L. et al. Rich-Club organization disturbances of the individual morphological network in subjective cognitive decline. *Front. Aging Neurosci.* **14**, 834145 (2022). 35283748 10.3389/fnagi.2022.834145 PMC8914315

[95] Li, Y. et al. Surface-based single-subject morphological brain networks: effects of morphological index, brain parcellation and similarity measure, sample size-varying stability and test-retest reliability. *NeuroImage* **235**, 118018 (2021). 33794358 10.1016/j.neuroimage.2021.118018

[96] Lin, J. Divergence measures based on the Shannon entropy. *IEEE Trans. Inf. theory* **37**, 145–151 (1991).

[97] Abadi, M. et al. {TensorFlow}: a system for {Large-Scale} machine learning. in *Proc*. *12th USENIX Symposium on Operating Systems Design and Implementation (OSDI 16)* (USENIX Association, 2016).

[98] Fornito, A. & Bullmore, E. T. Connectomics: a new paradigm for understanding brain disease. *Eur. Neuropsychopharmacol.* **25**, 733–748 (2015). 24726580 10.1016/j.euroneuro.2014.02.011

[99] Pineda, J. et al. Geometric deep learning reveals the spatiotemporal features of microscopic motion. *Nat. Mach. Intell.* **5**, 71–82 (2023).

[100] Midtvedt, B. et al. *Deep Learning Crash Course* (No Starch Press, 2025).

[101] Velickovic, P. et al. Graph attention networks. *stat* **1050**, 10–48550 (2017).

[102] Abdi, H. & Williams, L. J. Partial least squares methods: partial least squares correlation and partial least square regression. *Comput. Toxicol.* **II**, 549–579 (2013). 10.1007/978-1-62703-059-5_23 23086857

[103] de Lange, A. M. G. et al. Mind the gap: performance metric evaluation in brain-age prediction. *Hum. Brain Mapp.* **43**, 3113–3129 (2022). 35312210 10.1002/hbm.25837 PMC9188975

[104] Beheshti, I. et al. Bias-adjustment in neuroimaging-based brain age frameworks: a robust scheme. *NeuroImage Clin.* **24**, 102063 (2019). 31795063 10.1016/j.nicl.2019.102063 PMC6861562

[105] Cole, J. H. Multimodality neuroimaging brain-age in UK biobank: relationship to biomedical, lifestyle, and cognitive factors. *Neurobiol. Aging* **92**, 34–42 (2020). 32380363 10.1016/j.neurobiolaging.2020.03.014 PMC7280786

[106] Xiong, M. et al. Comparison of machine learning models for brain age prediction using six imaging modalities on middle-aged and older adults. *Sensors* **23**, 3622 (2023). 37050682 10.3390/s23073622 PMC10098634

[107] More, S. et al. Brain-age prediction: a systematic comparison of machine learning workflows. *NeuroImage* **270**, 119947 (2023). 36801372 10.1016/j.neuroimage.2023.119947

