# Supplementary material for: Similar minds age alike: an MRI similarity approach for predicting age-related cognitive decline
Source: NPJ Aging. 2026 Feb 6;12(1):39. doi: 10.1038/s41514-026-00345-1 (PMC12988147; doi:10.1038/s41514-026-00345-1)
Supplement: Supplementary file 1 — Supplementary information [file 41514_2026_345_MOESM1_ESM.docx]

**Supplementary Information - Similar Minds Age Alike: An MRI Similarity Approach for Predicting Age-Related Cognitive Decline**

**FIGURES**


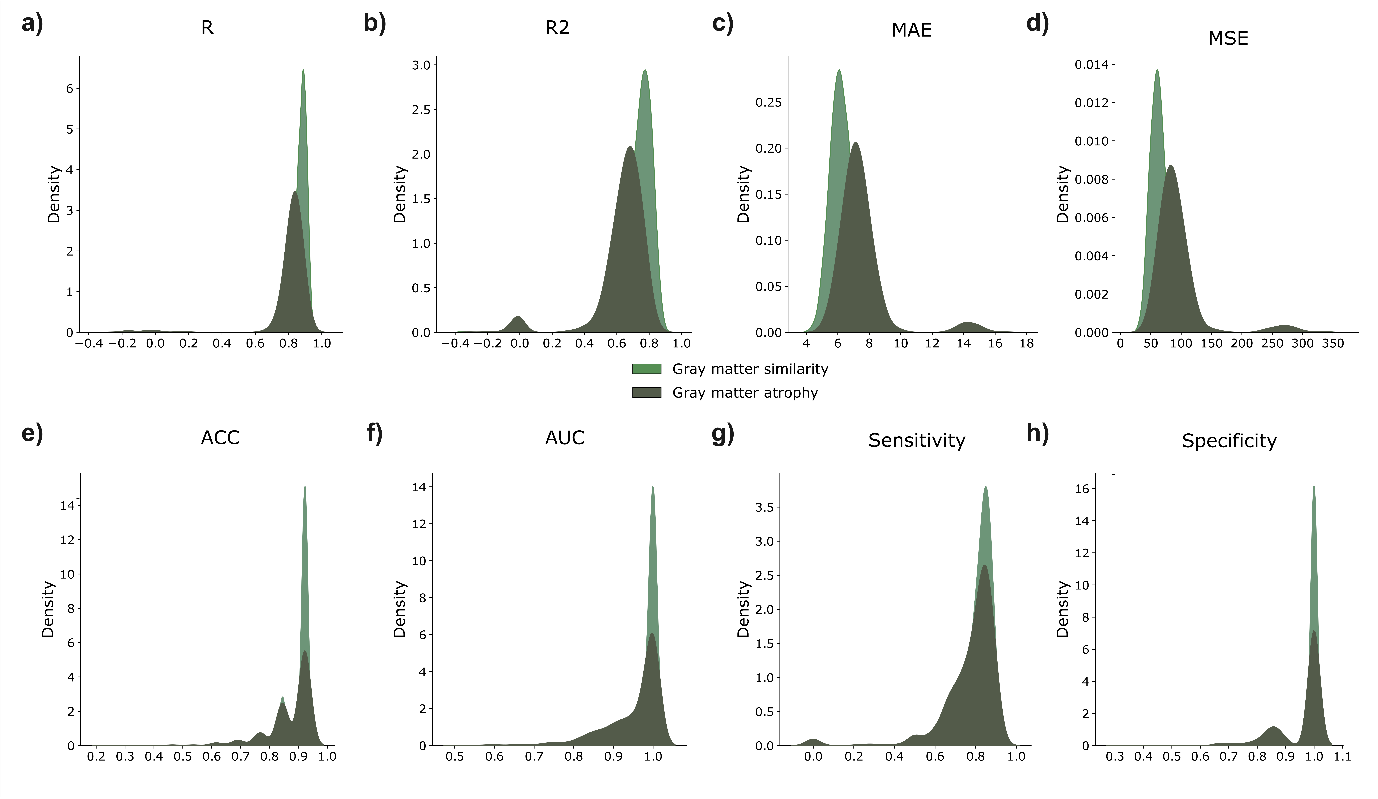


**Supplementary Figure 1. Performance metrics from brain similarity and atrophy models.** Kernel Density Estimate (KDE) plots display performance metrics across two different models: a graph neural network (GNN) model from gray matter similarity networks and a multi-layer perceptron (MLP) model from gray matter volumes. The brain similarity model achieves the highest performance in all the evaluations metrics for both Cam-CAN—a) Pearson Correlation Coefficient (R), b) Coefficient of Determination (R2), c) Mean Absolute Error (MAE) and d) Mean Square Error (MSE) between the true and predicted ages—and LEMON— e) Accuracy (ACC), f) Area Under the Curve (AUC), g) Sensitivity and h) Specificity—cohorts.


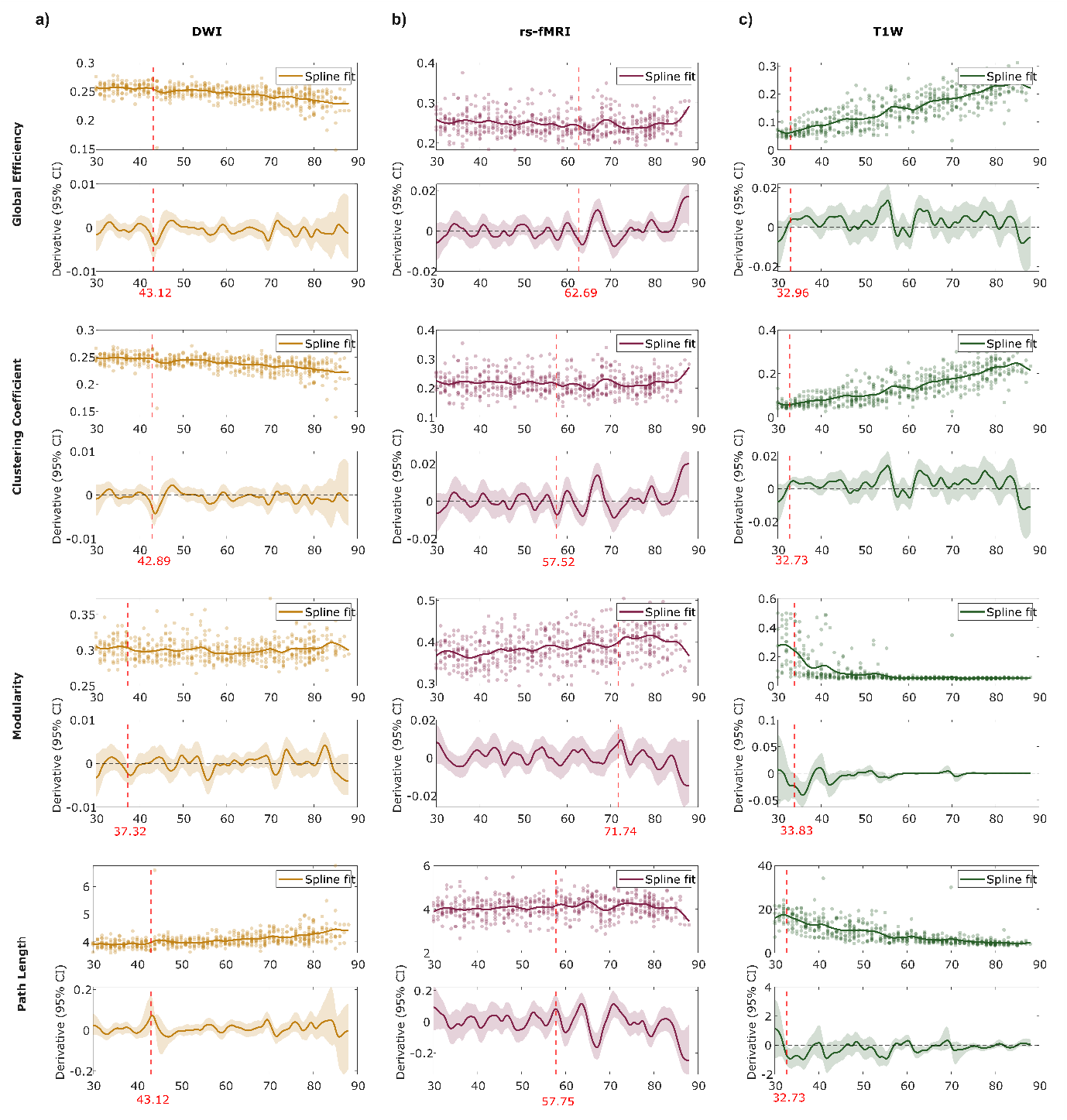


**Supplementary Figure 2.** **Changes of network measures across different age groups for the different types of networks.** a) Measures are calculated on a) anatomical connectivity derived from diffusion weighted imaging (DWI), b) functional connectivity derived from resting-state functional magnetic resonance imaging (rs-fMRI) and c) gray matter similarity networks computed from T1W imaging. Scatter plots are fitted with a spline model showing the relation between graph measures and age. Below the scatter plots, the first derivative of the spline model is shown with shaded areas representing the 95% confidence intervals. Early breakpoint ages are shown in red where brain similarity networks show the earliest age-related changes, followed by anatomical networks and functional networks.


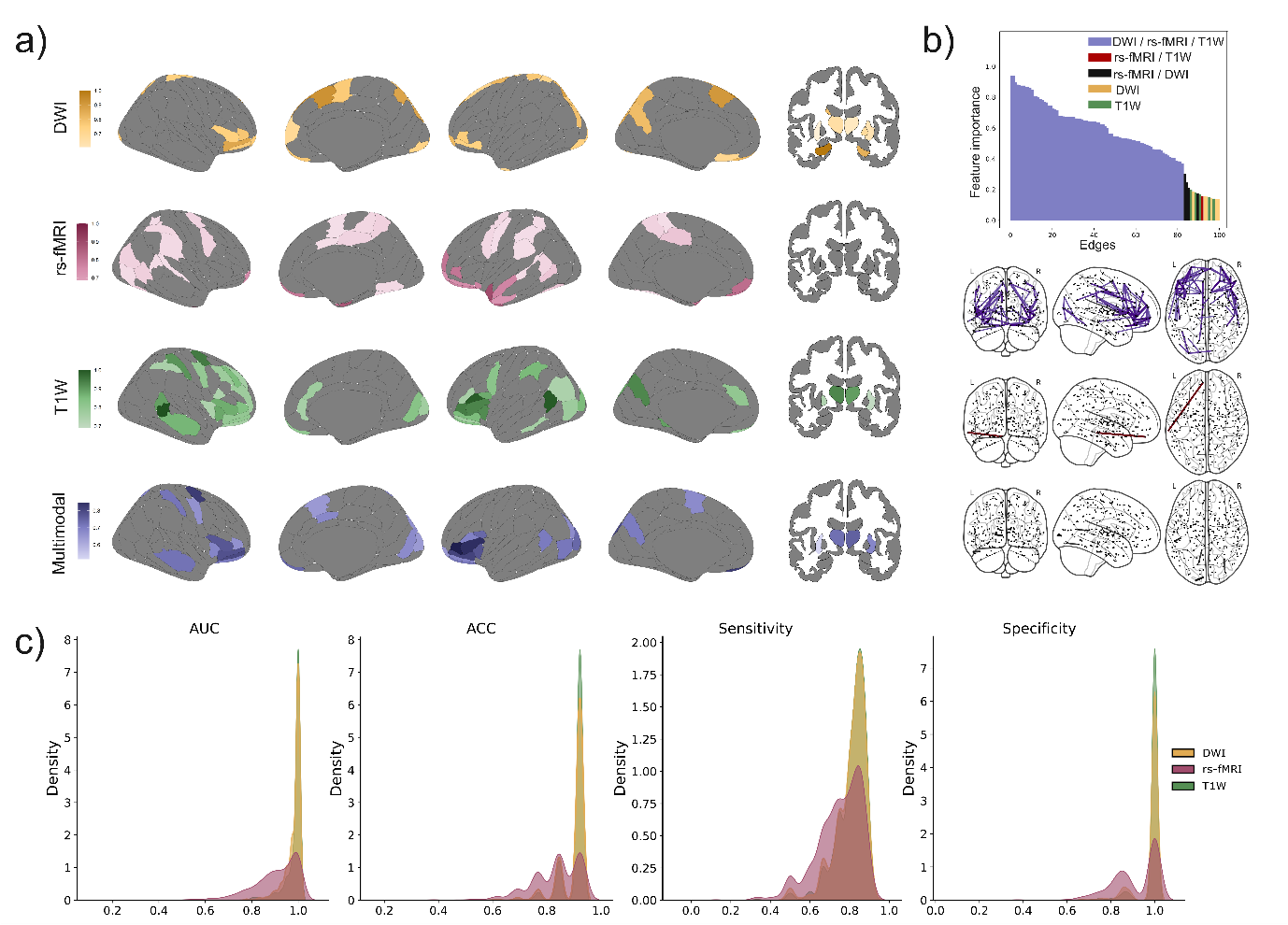


**Supplementary Figure 3.** **Results from the trained GNN on the different modalities in the LEMON cohort.** a) Top 20% regions that contributed the most to the prediction of age for the four different Graph Neural Network models: anatomical model derived from diffusion-weighted imaging (DWI), functional model derived from resting-state functional MRI (rs-fMRI), brain similarity model (T1W) and multimodal model (combining DWI, rs-fMRI and T1W). b) Edge feature importance from the multimodal model for the 100 most contributing edges. The distinct patterns of overlapping rs-fMRI-T1W (inter-lobe) and rs-fMRI -DWI (intra-lobe) connections provide further insights into the brain similarity-functional and anatomical-functional relationships. c) Kernel Density Estimate (KDE) plots display performance metrics across different models: Coefficient of Determination (R2), Pearson Correlation Coefficient (R), Mean Absolute Error (MAE) and Mean Square Error (MSE) between the true and predicted ages. The brain similarity model achieves the highest performance.


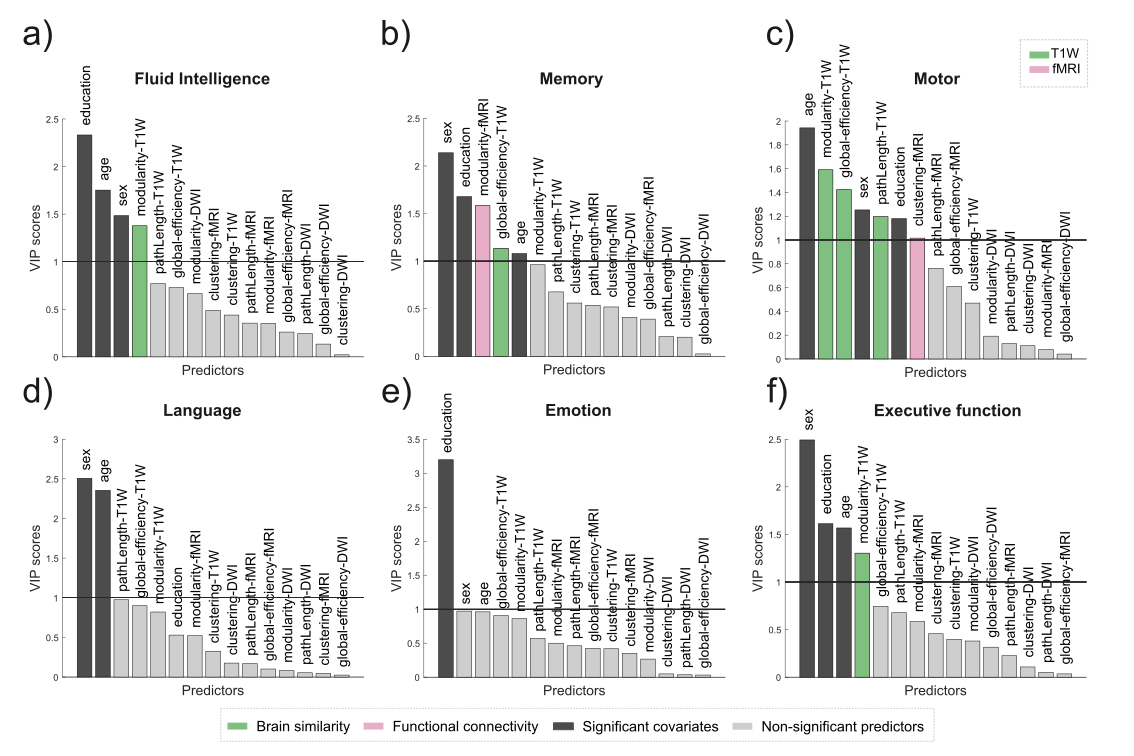


**Supplementary Figure 4.** **Findings on the association between the different types of networks and cognition and behavior in the LEMON cohort.** An independent Partial Least Square (PLS) model is computed for each of the following cognitive and behavioral tests: a) Fluid intelligence, b) Memory, c) Motor Control, d) Language, e) Emotion regulation and f) Executive function. Four network measures (global efficiency, path length, clustering coefficient, and modularity) derived from diffusion weighted imaging (DWI), resting-state functional MRI (rs-fMRI), and brain similarity (T1W) networks together with age, sex and education are included as predictors in the model. Significant predictors, selected if the variable of importance (VIP) is higher than one, are colored filled while non-significant predictors (VIP<1) are colored filled in gray. Network measures calculated on brain similarity networks emerged as the most significant predictors (colored in green), consistent with the primary analysis. However, functional connectivity measures gained higher importance in this cohort (colored in pink), emerging as significant predictors in memory and motor assessments.

*
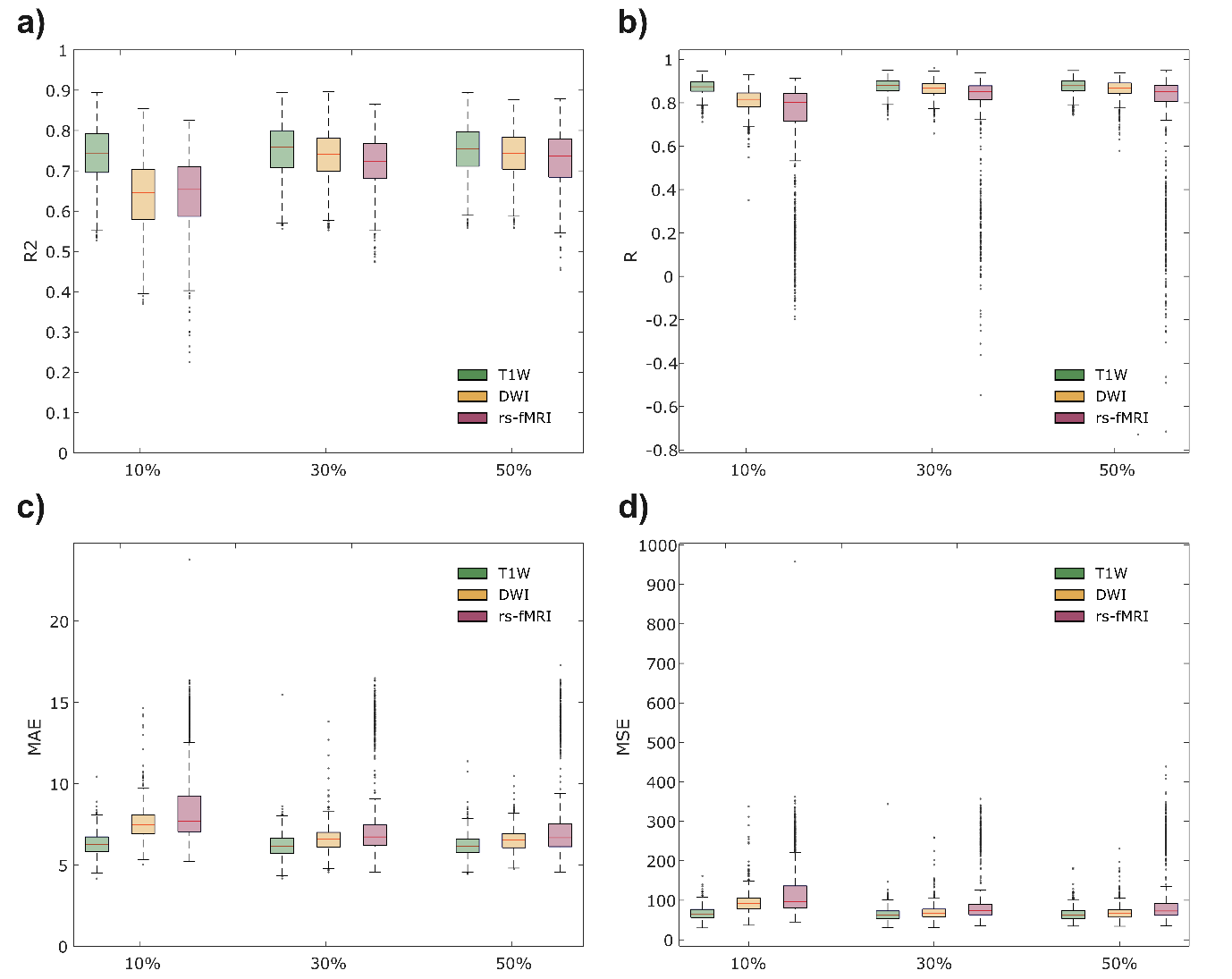
*

**Supplementary Figure 5. Performance of the GNN models evaluated using input networks thresholded at different densities (10%, 30%, and 50%).**We evaluated the three different Graph Neural Network models: anatomical model derived from diffusion-weighted imaging (DWI), functional model derived from resting-state functional MRI (rs-fMRI) and brain similarity model (T1W) across different densities (10%, 30% ad 50%). Model performance was evaluated across the different density thresholds using the coefficient of determination (R²), Pearson correlation coefficient (R), mean absolute error (MAE), and mean squared error (MSE) between true and predicted ages. Across all density levels, the brain similarity model consistently achieved the highest performance.

**TABLES**

| **LEMON cohort variables** | **N** | **Median (Min-Max)** |
| --- | --- | --- |
| **Age (years)** | 201 | 55 (39–71) |
| **Sex (Male/Female)** | (71/130) | - |
| **Education (years)** | 201 | 15 (17–23) |
| **Fluid Intelligence–Regensburger Wortflüssigkeits Test (RWT)** | 201 | 440 (272–919) |
| **Memory–California Verbal Learning Test (CVLT)** | 200 | 57.5 (2.5–94) |
| **Language–Vocabulary** | 194 | 4.66 (1.33–77) |
| **Emotion Regulation Questionnaire** | 201 | 19 (7–32) |
| **Motor & Action–Test battery for Attention Assessment (alertness)** | 200 | 554.5 (94–1244) |
| **Executive function–Trail Making Test (TMT)** | 194 | 20 (12–28) |

**Supplementary Table 1.** **Summary of characteristics and cognitive domain tests for LEMON cohort.** The sample size (N) and the median (minimum and maximum) values are displayed for each variable. Each participants underwent a series of tests intended to evaluate different cognitive and behavioral domains: fluid intelligence (Regensburger Wortflüssigkeits Test), memory (California Verbal Learning Test), language (Vocabulary), emotion regulation (Emotion Regulation Questionnaire), motor and action control (Test battery for Attention Assessment), and executive function (Trail Making Test).

| Modality | AUC | ACC | Sensitivity | Specificity |
| --- | --- | --- | --- | --- |
| DWI | 0.98 + 0.035 | 0.89+ 0.049 | 0.80 + 0.071 | 0.98 + 0.058 |
| rs-fMRI | 0.91 + 0.089 | 0.84+ 0.091 | 0.74 + 0.113 | 0.91 + 0.10 |
| T1W | **0.98 + 0.035** | **0.90 + 0.041** | **0.81 + 0.078** | **0.99 + 0.045** |
| DWI—rs-fMRI—T1W | 0.95 ± 0.108 | 0.87 ± 0.101 | 0.78 ± 0.118 | 0.96 ± 0.111 |

**Supplementary Table 2. Performance metrics across different types of models in the LEMON cohort.** Mean and standard deviation values of the following performance metrics: Coefficient of Determination (R2), Pearson Correlation Coefficient (R), Mean Absolute Error (MAE) and Mean Square Error (MSE) between the true and predicted ages. The metrics— Accuracy (ACC), Area Under the Curve (AUC), Sensitivity and Specificity—are calculated on test datasets for the 10-fold cross validation over 100 random iterations for the different types of models: diffusion-weighted imaging (DWI), resting-sate functional MRI (rs-fMRI), T1W and multimodal. Results highlighted in bold show significant (P<0.05) best performance.
